# 5-HT_1F_ Receptor Agonist Ameliorates Mechanical Allodynia in Neuropathic Pain via Induction of Mitochondrial Biogenesis and Suppression of Neuroinflammation

**DOI:** 10.3389/fphar.2022.834570

**Published:** 2022-03-03

**Authors:** Long-Qing Zhang, Ya-Qun Zhou, Jia-Yan Li, Jia Sun, Shuang Zhang, Jia-Yi Wu, Shao-Jie Gao, Xue-Bi Tian, Wei Mei

**Affiliations:** Department of Anesthesiology and Pain Medicine, Tongji Hospital, Tongji Medical College, Huazhong University of Science and Technology, Wuhan, China

**Keywords:** neuropathic pain, oxidative stress, mitochondrial dysfunction, mitochondrial biogenesis, 5-HT 1F receptor, PGC-1α

## Abstract

Neuropathic pain is a devastating disease that affects millions of people worldwide. Serotonin (5-hydroxytryptamine, 5-HT) is involved in pain modulation. Several lines of evidence have indicated that 5-HT_1F_ receptor agonists are potent inducers of mitochondrial biogenesis. In this study, we tested the hypothesis that 5-HT_1F_ receptor agonists ameliorate mechanical allodynia in neuropathic pain via the induction of mitochondrial biogenesis and suppression of neuroinflammation. Male Sprague–Dawley rats were used to establish a neuropathic pain model via spared nerve injury (SNI). The paw withdrawal threshold (PWT) was used to evaluate mechanical allodynia. Real-time polymerase chain reaction was used to examine the mitochondrial DNA (mtDNA) copy number. Western blotting and immunofluorescence were used to examine the expression of target proteins. Our results showed that mitochondrial biogenesis was impaired in the spinal cord of rats with SNI. Moreover, activation of PGC-1α, the master regulator of mitochondrial biogenesis, attenuates established mechanical allodynia in rats with neuropathic pain. In addition, the neuronal 5-HT_1F_ receptor is significantly downregulated in the spinal cord of rats with neuropathic pain. Furthermore, the selective 5-HT_1F_ receptor agonist lasmiditan attenuated established mechanical allodynia in rats with neuropathic pain. Finally, lasmiditan (Las) treatment restored mitochondrial biogenesis and suppressed neuroinflammation in the spinal cord of rats with SNI. These results provide the first evidence that lasmiditan ameliorates mechanical allodynia in neuropathic pain by inducing mitochondrial biogenesis and suppressing neuroinflammation in the spinal cord. Inducers of mitochondrial biogenesis may be an encouraging therapeutic option for the management of neuropathic pain.

## Introduction

Chronic neuropathic pain is a devastating disease that results from damage to or dysfunction of the nervous system (Scholz et al., 2019). It affects millions of people worldwide and severely disrupts their quality of life. Unfortunately, it is refractory to commonly used analgesics such as nonsteroidal anti-inflammatory drugs and opioids (Stacey et al., 2008). Tricyclic antidepressants and antiepileptics also fail to provide sufficient pain relief. Despite rapid advances over the past few decades, the specific cellular and molecular mechanisms underlying the development of neuropathic pain remain largely unknown (Ge et al., 2020; Ruiz-Cantero et al., 2020). Therefore, there is an urgent need to elucidate the pathogenesis of neuropathic pain and identify novel therapeutic targets.

Emerging evidence from our laboratory and others indicates that oxidative stress and mitochondrial dysfunction may be the primary causes of chronic pain (Zhou et al., 2018; Trecarichi and Flatters, 2019; Zhou et al., 2020b). Mitochondrial biogenesis, a dynamic process that generates new mitochondria, plays a critical role in regulating mitochondrial homeostasis (Li et al., 2017; Li Y. et al., 2020). Peroxisome proliferator-activated receptor γ coactivator 1α (PGC-1α) is a master regulator of mitochondrial biogenesis (Popov 2020; Rius-Perez et al., 2020). PGC-1α activates nuclear respiratory factor 1 (NRF1) and, subsequently, mitochondrial transcription factor A (TFAM). Impaired mitochondrial biogenesis has been reported to contribute to mitochondrial dysfunction in neurological diseases such as Alzheimer’s disease and spinal cord injury (Sheng et al., 2012; Scholpa et al., 2019). Moreover, a recent study has demonstrated that PGC-1α haploinsufficiency promotes pain chronification after burn injury (Miao et al., 2020). Furthermore, our recent studies have indicated that activation of PGC-1α attenuates neuropathic pain (Chen et al., 2021; Sun et al., 2021). Additionally, recent evidence has indicated that PGC-1α activation exerts neuroprotective effects by suppressing neuroinflammation (Fu et al., 2020; Han et al., 2021). Given the vital role of neuroinflammation in chronic pain (Song et al., 2017; Zhang et al., 2017), it is plausible that PGC-1α activation might attenuate pain behaviors in neuropathic pain via the suppression of neuroinflammation.

Serotonin (5-hydroxytryptamine, 5-HT), a monoamine widely distributed in the central nervous system, is involved in pain modulation (Bravo et al., 2019). To date, seven 5-HT receptor families have been identified. Previous studies have demonstrated that several 5-HT receptor subtypes mediate 5-HT-induced antinociception such as 5-HT_5A_ receptor and 5-HT_7_ receptor (Brenchat et al., 2010; Munoz-Islas et al., 2014). However, little is known regarding the role of 5-HT_1F_ receptors in neuropathic pain. Although the physiological role of 5-HT_1F_ has not been fully characterized, it is expressed in the spinal cord and various brain regions involved in pain (Bruinvels et al., 1994; Simmons et al., 2020). Moreover, LY344864, a 5-HT_1F_ receptor agonist, attenuated formalin-induced inflammatory pain (Granados-Soto et al., 2010). Lasmiditan, a highly selective 5-HT_1F_ receptor agonist, has been approved by the United States Food and Drug Administration for acute treatment of migraine (Lamb, 2019). Furthermore, several lines of evidence have indicated that 5-HT_1F_ receptor agonists are potent inducers of mitochondrial biogenesis (Rasbach et al., 2010; Garrett et al., 2014; Scholpa et al., 2018; Dupre et al., 2019). Moreover, the 5-HT_1F_ receptor is expressed in the dorsal root ganglion at the lumbar level (Classey et al., 2010), and activation of the 5-HT_1F_ receptor with LY344864 attenuates formalin-induced inflammatory pain (Granados-Soto et al., 2010). Several studies have indicated that mitochondrial dysfunction may be the primary cause of chronic pain. Mitochondrial biogenesis plays an important role in maintaining mitochondrial function (Bi et al., 2019). Furthermore, impairment of mitochondrial biogenesis contributes to the development of chronic pain (Chen et al., 2021). Moreover, activation of the 5-HT_1F_ receptor by LY344864 activates PGC-1α and promotes mitochondrial biogenesis in Parkinson’s disease (PD) mice (Scholpa et al., 2018). There may be crosstalk between the 5-HT_1F_ receptor and mitochondrial biogenesis during neuropathic pain. Given the crucial role of mitochondrial dysfunction in neuropathic pain and the involvement of 5-HT_1F_ receptors in mitochondrial biogenesis, this study aimed to test the hypothesis that 5-HT_1F_ receptor agonists ameliorate mechanical allodynia in neuropathic pain via the induction of mitochondrial biogenesis and suppression of neuroinflammation.

## Materials and Methods

### Animals and Ethical Statement

In total, 496 rats were used in this study. Adult male Sprague–Dawley rats (180–220 g) were supplied by Tongji Hospital, Tongji Medical College, Huazhong University of Science and Technology (Wuhan, China). The rats were housed under controlled conditions (temperature: 22–25°C, relative humidity: 45–65%, and 12-h light to dark cycle, with food and water ad libitum). All the experiments were approved by the Experimental Animal Care and Use Committee of Tongji Hospital, Tongji Medical College, Huazhong University of Science and Technology. All experiments were conducted in accordance with the National Institutes of Health Guidelines for the Care and Use of Laboratory Animals and ARRIVE Guidelines for Reporting Animal Research (Percie du Sert et al., 2020). All efforts were made to minimize the number of animals used and their suffering.

### Establishment of Neuropathic Pain Model

A rat model of neuropathic pain was induced by SNI as described previously (Decosterd. and Woolf, 2000; Xiong et al., 2020). Briefly, rats were anesthetized with 2.5% isoflurane, and the right sciatic nerve and its three branches (the common peroneal, tibial, and sural nerves) were exposed. The common peroneal and tibial nerves were then ligated and excised. For sham-surgery rats, the sciatic nerve was exposed without ligation.

### Intrathecal Catheterization and Drug Administration

As described previously (Zhou et al., 2017), intrathecal (i.t.) catheterization was performed 5 days prior to the establishment of SNI models. Briefly, rats were anesthetized using 2.5% isoflurane. Then, PE10 polyethylene catheters (inner diameter 0.3 mm, outer diameter 0.6 mm) were inserted from the L5–L6 spinous processes. The correct position of the catheter was verified by a tail-flick response immediately after insertion and further confirmed using an i. t. injection of 2% lidocaine.

Lasmiditan succinate (Cat# S6489, Selleckchem, Houston, TX, United States), a selective 5-HT_1F_ receptor agonist, was dissolved in saline. ZLN005 (Cat# HY-17538, MedChemExpress, Monmouth Junction, NJ, United States), a specific PGC-1α activator, was dissolved in 10% dimethyl sulfoxide (DMSO). SR-18292 (Cat# HY-101491, MedChemExpress), a PGC-1α inhibitor, was dissolved in 10% DMSO. In this study, we investigated a purely spinal mechanism. Therefore, we chose intrathecal rather than intraperitoneal or oral injections. The dosages of these drugs were determined in preliminary experiments. To determine whether a single dose of PGC-1α activator or 5-HT_1F_ receptor agonist could attenuate established mechanical allodynia in SNI rats, ZLN005 (10, 50, or 100 μg, i. t.) or Lasmiditan (50, 100, or 200 μg, i. t.) was administered on day 7 after SNI. Behavioral tests were conducted before ZLN005 or lasmiditan injection and 1, 2, 4, 6, and 12 h after the injection. To determine whether repeated injection of a PGC-1α activator or 5-HT_1F_ receptor agonist reversed mechanical allodynia in SNI rats, ZLN005 (50 μg, i. t.) or lasmiditan (100 μg, i. t.) was administered once daily for five consecutive days starting from day 7. The behavioral test was performed on day 6 and 1 h after ZLN005 injection or 2 h after lasmiditan injection. To determine whether early treatment with a PGC-1α activator or 5-HT_1F_ receptor agonist could suppress the development of mechanical allodynia in SNI rats, ZLN005 (50 μg, i. t.) or lasmiditan (100 μg, i. t.) was administered once daily for 7 consecutive days starting from day 1 after the surgery. The behavioral test was conducted before surgery and on days 1, 3, 7, 8, 9, 10, 11, 12, 13, and 14 after surgery. To determine whether a PGC-1α inhibitor could reverse the analgesic effects of ZLN005 or lasmiditan, SR-18292 (30 μg, i. t.) was administered 30 min before ZLN005 or lasmiditan. Behavioral tests were conducted before SR-18292 injection and 1, 2, 4, 6, and 12 h after ZLN005 or lasmiditan injection. To determine whether the 5-HT_1F_ receptor agonist affected the PWT in naïve rats, lasmiditan (100 μg, i. t.) was administered once daily for five consecutive days. The behavioral test was performed 2 h after the lasmiditan injection each day. Syringes containing various drugs were randomly coded by another researcher. Drugs in the syringes were injected into the rats according to the codes.

### Behavioral Tests

To measure mechanical allodynia, the PWT in response to von Frey filament stimuli was assessed as described previously (Chen et al., 2018). Briefly, the rats were placed in individual plastic boxes on a metal mesh floor and allowed to habituate for 30 min. Von Frey filaments (2, 4, 6, 8, 10, and 15 g) were applied for up to 6 s per filament to the mid-plantar region of the right hind paw. A positive response was defined as a sudden paw withdrawal, licking, or shaking. In case of a positive response, the paw was retested after a 5-min rest, starting with the next descending von Frey filament. If there was no response, the next higher filament was applied. The lowest amount of force required to elicit a positive response was recorded as the PWT (in grams). The results are shown in log PWT. All behavioral tests were performed by an investigator who was blinded to the experimental design.

### Western Blotting

Antibody-based procedures complied with the recommendations of the British Journal of Pharmacology (Alexander et al., 2018). The rats were anesthetized with 2.5% isoflurane. The L4–6 spinal cord was immediately removed and homogenized in an ice-cold mixture of radioimmunoprecipitation assay lysis buffer (Cat# AR0102; Boster Biological Technology, Wuhan, Hubei, China), phosphatase inhibitor (Cat# AR1183; Boster Biological Technology), and phenylmethylsulfonyl fluoride (Cat# AR1178; Boster Biological Technology), and then centrifuged at 12,000 rpm at 4°C for 30 min. The supernatants were collected, and the protein concentration was determined using the Bradford method. The proteins were boiled at 95°C in a loading buffer for 10 min. Equivalent amounts of samples (30-μg protein) were separated by 10% sodium dodecyl sulfate-polyacrylamide gel electrophoresis and transferred onto polyvinylidene fluoride membranes (Cat# IPVH00010; Millipore, Billerica, MA, United States). After blocking with 5% bovine serum albumin in Tris-buffered saline and Tween 20 (0.1%) (TBST) for 2 h at room temperature (RT), the membranes were incubated overnight at 4°C with rabbit anti-5-HT_1F_ receptor antibody (1:500; Cat# DF3499; RRID:AB_2835861; Affinity; OH; United States); rabbit anti-PGC-1α antibody (1:1,000; Cat# ab54481; RRID:AB_881987; Abcam, Cambridge, United Kingdom); rabbit anti-NRF1 antibody (1:1,000; Cat# ab175932; RRID:AB_2629496; Abcam); rabbit anti-TFAM antibody (1:500; Cat# ab131607, RRID:AB_11154693; Abcam); mouse anti-glial fibrillary acidic protein (GFAP, astrocytic marker) antibody (1:500; Cat# 3670; RRID:AB_561049; Cell Signaling Technology, Danvers, MA, United States); goat anti-ionized calcium-binding adapter molecule 1(Iba-1, microglial marker) antibody (1:500; Cat# ab5076; RRID:AB_2224402; Abcam); rabbit-anti interleukin-1 beta antibody (IL-1β; 1:1,000; Cat# AF4006; RRID:AB_2801567; Affinity); rabbit anti-tumor necrosis factor alpha antibody (TNF-α,1:500; Cat# ab205587; RRID:AB_2889389; abcam); mouse anti-IL-6 antibody (1:500; Cat# A0286; RRID:AB_2757098; ABclonal, Woburn, MA, United States); mouse anti-glyceraldehyde-3-phosphate dehydrogenase antibody (GAPDH) (1:5,000; Cat# AC033; RRID:AB_2769570; ABclonal), respectively. The membranes were then washed in TBST and incubated with horseradish peroxidase-conjugated goat anti-rabbit (1:5,000; Cat# A21020; RRID:AB_2876889; Abbkine, Wuhan, Hubei, China), goat anti-mouse secondary antibody (1:5,000; Cat# A21010; RRID:AB_2728771; Abbkine), or donkey anti-goat antibody (1:5,000; Cat# AS031; RRID:AB_2769846; ABclonal) for 2 h at RT. The bands were finally visualized with the SuperLumia ECL Plus HRP Substrate Kit (Cat# K22030; Abbkine) and then detected using a computerized image analysis system (Bio-Rad, ChemiDoc XRS+, United States). The intensity of protein blots was quantified using the System with Image Lab software (Bio-Rad Laboratories), normalized to the loading control GAPDH antibody, and expressed as the fold of control. The blot density of the naïve group was set to 1. The original data were coded by one researcher, the different codes represented different groups, and the data were analyzed by another researcher.

### Immunofluorescence

Antibody-based procedures complied with the recommendations of the British Journal of Pharmacology (Alexander et al., 2018). Under deep anesthesia with 2.5% isoflurane, the rats were perfused intracardially with 0.1 M phosphate buffered saline (PBS), followed by 4% ice-cold paraformaldehyde (PFA) in PBS. Then, the L4–6 spinal cord was removed and post-fixed in 4% PFA for 4 h and subsequently dehydrated in 30% sucrose solution overnight at 4°C. The collected spinal cord samples were sectioned to 20-μm thickness using a cryostat (CM 1900, Leica, Wetzlar, Germany). For single immunofluorescence, the sections were blocked with 5% goat serum or donkey serum (for detecting the expression of Iba-1) and 0.3% Triton X-100 for 1 h at RT, and then incubated with rabbit anti-5HT_1F_ receptor antibody (1:50; Cat# DF3499; RRID:AB_2835861; Affinity), mouse anti-GFAP antibody (1:100; Cat# 3,670; RRID:AB_561,049; Cell Signaling Technology), or goat anti-Iba-1 antibody (1:50; Cat# ab5076; RRID:AB_2224402; Abcam) overnight at 4°C. After washing three times in PBS, the sections were incubated with Alexa Fluor 594-labeled goat anti-rabbit secondary antibody (1:300; Cat# 111-585-003; RRID:AB_2338059; Jackson ImmunoResearch, West Grove, PA, United States), Alexa Fluor 488-labeled goat anti-mouse secondary antibody (1:200; Cat# 115-545-003; RRID:AB_2338840; Jackson ImmunoResearch), or FITC-conjugated AffiniPure donkey anti-goat secondary antibody (1:50; Cat# SA00003-3; RRID:AB_2857365; Proteintech, Chicago, IL, United States) for 2 h at RT. For double immunofluorescence, the sections were blocked with 5% goat serum and 0.3% Triton X-100 for 1 h at RT, and then incubated with a mixture of rabbit anti-5HT_1F_ receptor antibody (1:50; Cat# DF3499; RRID:AB_2835861; Affinity) and mouse anti-GFAP antibody (1:100; Cat# 3,670; RRID:AB_561049; Cell Signaling Technology), mouse anti-neuronal nuclei (NeuN, neuronal marker) antibody (1:50; Cat# ab104224; RRID:AB_10711040; Abcam), mouse anti-calcitonin gene-related peptide antibody (CGRP, 1:100; Cat# ab81887, RRID:AB_1658411; Abcam), or FITC-conjugated isolectin B4 (IB4, 1:100; Cat# L2140; RRID: AB_2313663; Sigma-Aldrich; St. Louis, Missouri, United States). The sections were then incubated with a mixture of secondary antibodies, including Alexa Fluor 594-labeled goat anti-rabbit secondary antibody (1:300; Cat# 111-585-003; RRID:AB_2338059; Jackson ImmunoResearch) and Alexa Fluor 488-labeled goat anti-mouse secondary antibody (1:200; Cat# 115-545-003; RRID:AB_2338840; Jackson ImmunoResearch) for 2 h at RT. The sections were blocked with 5% donkey serum and 0.3% Triton X-100 for 1 h at RT and then incubated with a mixture of rabbit anti-5HT_1F_ receptor antibody (1:50; Cat# DF3499; RRID:AB_2835861; Affinity) and goat anti-Iba-1antibody (1:50; Cat# ab5076; RRID:AB_2224402; Abcam). The sections were then incubated with a mixture of secondary antibodies, including CoraLite 594-conjugated donkey anti-rabbit secondary antibody (1:100; Cat# SA00013-8; RRID:AB_2857367; Proteintech) and FITC-conjugated AffiniPure donkey anti-goat secondary antibody (1:50; Cat# SA00003-3; RRID:AB_2857365; Proteintech). Images were captured using a fluorescence microscope (BX51, Olympus, Japan). To measure the mean intensity of the 5-HT_1F_ receptor, NeuN, GFAP, or Iba-1, three sections from each animal were evaluated. As previously described, the mean fluorescence intensity was calculated using ImageJ (National Institutes of Health, Bethesda, MD, United States) (Li T. et al., 2020; Wu et al., 2020). The average fluorescence intensity of each pixel was normalized to the background intensity of the same image. To measure the ratio of colocalization in the dorsal horn, the percentage of overlapped pixels (5-HT_1F_R^+^/marker^+^) from the total marker^+^ pixels was used to represent the degree of colocalization. All images were analyzed by an investigator who was blinded to the experimental design. The original data were coded by one researcher, the different codes represented different groups, and the data were analyzed by another researcher.

### Absolute Quantification of mtDNA Copy Number

Under deep anesthesia with 2.5% isoflurane, the L4–6 spinal cord of the rat was removed. The DNA isolation from the spinal cord was performed according to the manufacturer’s protocol (DNO7; Aidlab Biotechnologies, Beijing, China) and stored at −20°C until use. The protocols for the absolute quantification of mtDNA copy number were as follows: First, we constructed a standard detection pUC57-mtDNA plasmid, according to the conserved sequence of mtDNA, and then the standard plasmid was transfected into DH5 α competent cells for further propagation and purification. Second, the concentration and DNA copy number of standard plasmids were determined according to the following equation: copies/μl= (6.02 × 10^23) × (plasmid concentration ng/μl × 10^-9)/(DNA length × 660). Third, the standard plasmids (DNA copy number) were diluted with DNase ddH_2_O and the range of dilutions were 10^6-10^0, and the diluted plasmids were used as templates for real-time polymerase chain reaction amplification (RT-PCR) (7900HT, Applied Biosystems; United States). Fourth, a standard curve (Y = -0.6388 × X+17.554, X = Ct, Y = log copy number) was constructed according to the results of RT-PCR. The PCR reaction system (20 μl) includes 4-μl DNA, 10-μl PCR mix, 1-μl mtDNA primer forward, 1-μl mtDNA primer reverse, and 4-μl ddH_2_O. The protocols for the PCR were as follows: pre-incubation at 95°C for 5 min (1 cycle); denaturation at 95°C for 10 s; annealing and extension at 58°C for 30 s and 72°C for 15 s (repeat denaturation and extension steps for 40 cycles); melting at 95°C for 15 s and 60°C for 60 s, (melt curve analysis: 1 cycle). The primers used were as follows: mtDNA forward: 5ʹ- CGC​CAG​GGT​TTT​CCC​AGT​CAC​GAC- 3ʹ, mtDNA reverse: 5ʹ- AGC​GGA​TAA​CAA​TTT​CAC​ACA​GGA- 3ʹ. Finally, the lumbar spinal cord samples were amplified using the same PCR system and the absolute mtDNA copy number was calculated using a standard curve. The original data were coded by one researcher, the different codes represented different groups, and the data were analyzed by another researcher.

### Experimental Design

All experiments were performed under blinded conditions. The rats were randomly assigned to different groups and allocated to groups using a Research Randomizer (https://www.randomizer.org/). The sample size of each group was determined according to the calculators (http://powerandsamplesize.com/Calculators/). The SNI model results in early (<24 h), prolonged (>6 months), and robust (all animals are responders) behavioral modifications, and the mechanical (von Frey) sensitivity is increased in the ipsilateral sural and, to a lesser extent, saphenous territories. Therefore, we chose SNI as a model of neuropathic pain in this study. None of the animals were excluded during the study period.

Experiment 1: To explore whether impaired mitochondrial biogenesis participates in the development of neuropathic pain, 108 rats were randomly assigned to six groups (n = 18 rats per group). When the final behavioral test was completed (we only showed the behavioral results of 10 rats in each group), the L4–6 vertebra lumbalis were collected for WB, IF, and quantification of mtDNA copy number.

Experiment 2: To determine whether a single dose of PGC-1α activator could attenuate established mechanical allodynia in rats with SNI, 40 rats were randomly assigned to four groups (n = 10 rats per group).

Experiment 3: To determine whether repeated injections of a PGC-1α activator agonist could reverse mechanical allodynia in SNI rats, 40 rats were randomly assigned to four groups (n = 10 rats per group).

Experiment 4: To determine whether early treatment with a PGC-1α activator could suppress the development of mechanical allodynia in rats with SNI, 40 rats were randomly assigned to four groups (n = 10 rats per group).

Experiment 5: To determine whether a PGC-1α inhibitor could reverse the analgesic effects of a PGC-1α activator, 40 rats were randomly assigned to four groups (n = 10 rats per group).

Experiment 6: To determine whether a single dose of a 5-HT1F receptor agonist could attenuate established mechanical allodynia in rats with SNI, 40 rats were randomly assigned to four groups (n = 10 rats per group).

Experiment 7: To determine whether repeated injections of a 5-HT1F receptor agonist could reverse mechanical allodynia in SNI rats, 72 rats were randomly assigned to four groups (n = 18 rats per group). When the final behavioral test was completed (we only showed the behavioral results of 10 rats in each group), the L4–6 spinal cord was collected for WB, IF, and quantification of mtDNA copy number.

Experiment 8: To determine whether early treatment with a 5-HT1F receptor agonist could suppress the development of mechanical allodynia in rats with SNI, 40 rats were randomly assigned to four groups (n = 10 rats per group).

Experiment 9: To determine whether a PGC-1α inhibitor could reverse the analgesic effects of a 5-HT1F receptor agonist, 40 rats were randomly assigned to four groups (n = 10 rats per group).

Experiment 10: To determine whether the 5-HT1F receptors agonist affects PWT in naïve rats, 36 rats were randomly assigned to three groups (n = 12 rats in each group). When the final behavioral test was completed (we only showed the behavioral results of 10 rats in each group), the L4–6 spinal cord was collected for WB and quantification of mtDNA copy number.

### Data and Statistical Analysis

The data and statistical analysis complied with the recommendations of experimental design and analysis in pharmacology (Curtis et al., 2018). All statistical analyses were performed blindly using these independent values. No data points were excluded and no additional data were subjected to statistical analysis in any experiment. Each animal provided an independent value and statistical analyses were performed using these numbers. All data are presented as the mean ± standard error of the mean (SEM) and were analyzed using GraphPad Prism version 6 (Graph Pad Software, San Diego, CA, United States). Data normality was verified using the Shapiro–Wilk test, except for the western blot results. Data derived from the Gaussian distribution were further tested for conformity with the ANOVA. Other variables were log-transformed or tested using non-parametric analyses. For western blotting, normalization was performed to compare the differences among groups, control for unwanted sources of variation, and reveal relevant trends. The protein level is presented as the density relative to that of GAPDH. Data are expressed as fold change relative to the sham group. Normalization removes the variance from the control values (SEM = 0). Data were subjected to Kruskal–Wallis test followed by Dunn’s post hoc test for non-parametric statistical analysis. In the figures, the “fold matched control” is indicated as the “relative protein densitometry.” mtDNA copy number data were analyzed using one-way analysis of variance (ANOVA) followed by Bonferroni’s post-hoc test for parametric analysis. The post hoc test was performed only if F was significant and there was no variance inhomogeneity. The behavioral test was analyzed by two-way repeated measures ANOVA (RM ANOVA; treatment group × time) to detect overall differences among treatment groups, followed by Bonferroni’s test to detect changes in log PWT after drug injection over time. Before the two-way RM ANOVA, the behavioral test data were analyzed using Mauchly’s sphericity test, followed by multivariate ANOVA (MANOVA). When the data did not violate the sphericity assumption (*p* > 0.05), the MANOVA F value was analyzed using the assumed degrees of freedom for sphericity. However, if the sphericity assumption was violated (*p* < 0.05), the F value was analyzed using Greenhouse–Geisser’s corrected degrees of freedom. When the F-value was significant, the data were analyzed using Bonferroni’s post-hoc test. Single immunofluorescence staining was analyzed using one-way ANOVA followed by Bonferroni’s post hoc test for parametric analysis. The post hoc test was performed only if F was significant and there was no variance inhomogeneity. Double immunofluorescence staining was analyzed using an unpaired Student’s t-test when the data did not violate the sample variance homogeneity assumption (*p* > 0.05). Statistical significance was set at *p* < 0.05.

## Results

### SNI-Induced Mechanical Allodynia and Mitochondrial Biogenesis Impairment in the Spinal Cord

**FIGURE 1:**
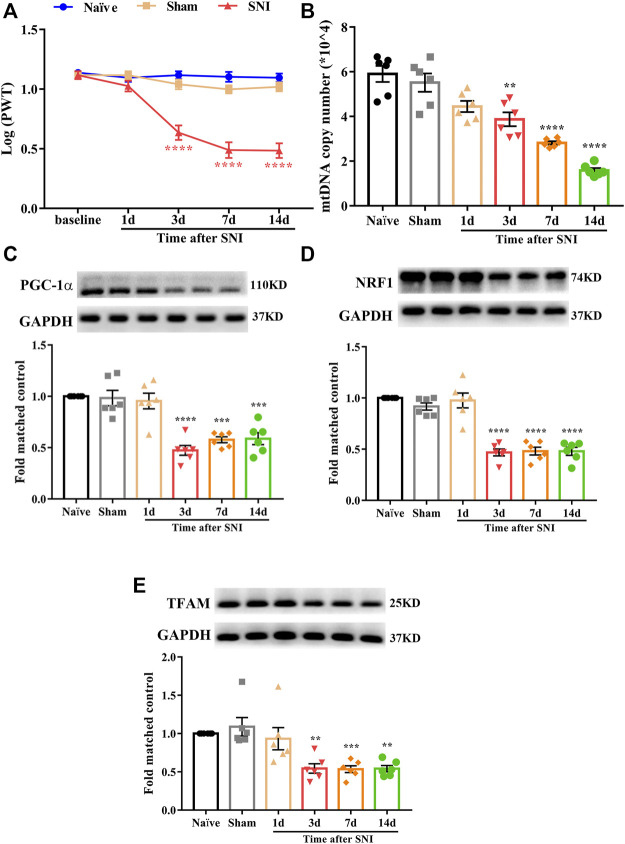
Mechanical allodynia induced by spared nerve injury and mitochondrial biogenesis was impaired in the spinal cord of neuropathic pain rats. **(A)** Paw withdrawal threshold (PWT) was assessed to evaluate the development of mechanical allodynia at baseline and 1, 3, 7, and 14 days after surgery. There is no significant difference regarding the PWT among naïve, sham, and SNI group at baseline. However, the PWT in SNI rats was markedly decreased from day 3 to day 14 (^****^ *p* < 0.0001 compared with sham group, n = 10 in each group). In contrast, the PWT in sham rats had no significant change during the observation period. **(B)** Mitochondrial DNA (mtDNA) copy number was significantly and time dependently decreased in SNI rats, beginning at day 3 after surgery and remain at low level until day 14 (^**^ *p* < 0.01, ^****^ *p* < 0.0001 compared with sham group, n = 6 in each group). **(C)** PGC-1α was significantly decreased in SNI rats, beginning at day 3 after surgery and remain at low level until day 14 (^***^ *p* < 0.001, ^****^ *p* < 0.0001 compared with sham group, n = 6 in each group). **(D)** NRF1 was significantly decreased in SNI rats, beginning at day 3 after surgery and remain at low level until day 14 (^****^ *p* < 0.0001 compared with sham group, n = 6 in each group). **(E)** TFAM was significantly decreased in SNI rats, beginning at day 3 after surgery and remain at low level until day 14 (^**^ *p* < 0.01, ^***^ *p* < 0.001 compared with sham group, n = 6 in each group).

In this study, we used a well-established rat model of neuropathic pain induced by SNIs. The PWT was assessed to evaluate the development of mechanical allodynia at baseline and 1, 3, 7, and 14 days after surgery. As shown in Figure 1A, there was no significant difference in the PWT among naïve, sham, and SNI rats at baseline. However, the PWT in SNI rats markedly decreased from day 3 to day 14 (^****^ *p* < 0.0001 compared to the sham group, n = 10 in each group). In contrast, the PWT in the sham rats showed no significant changes during the observation period. These results indicated that SNI successfully induced the development of mechanical allodynia.

To date, little is known regarding the role of mitochondrial biogenesis in neuropathic pain. First, we determined whether mitochondrial biogenesis was impaired in rats with SNI. The mtDNA copy number and protein level of PGC-1α, NRF1, and TFAM in the spinal cord were examined. As shown in Figure 1B, mtDNA copy number was significantly decreased in SNI rats, beginning at day 3 after surgery and remaining at a low level until day 14 (^**^ *p* < 0.01, ^****^ *p* < 0.0001 compared with the sham group, n = 6 in each group). Similarly, the protein level of PGC-1α, NRF1, and TFAM were significantly decreased in SNI rats (Figures 1C–E, ^**^ *p* < 0.01, ^***^ *p* < 0.001, ^****^ *p* < 0.0001 compared with the sham group, n = 6 in each group). These results suggest that mitochondrial biogenesis is impaired in the spinal cords of rats with SNI.

### Analgesic Effect of PGC-1α Activator on Mechanical Allodynia in Neuropathic Pain Rats

**FIGURE 2:**
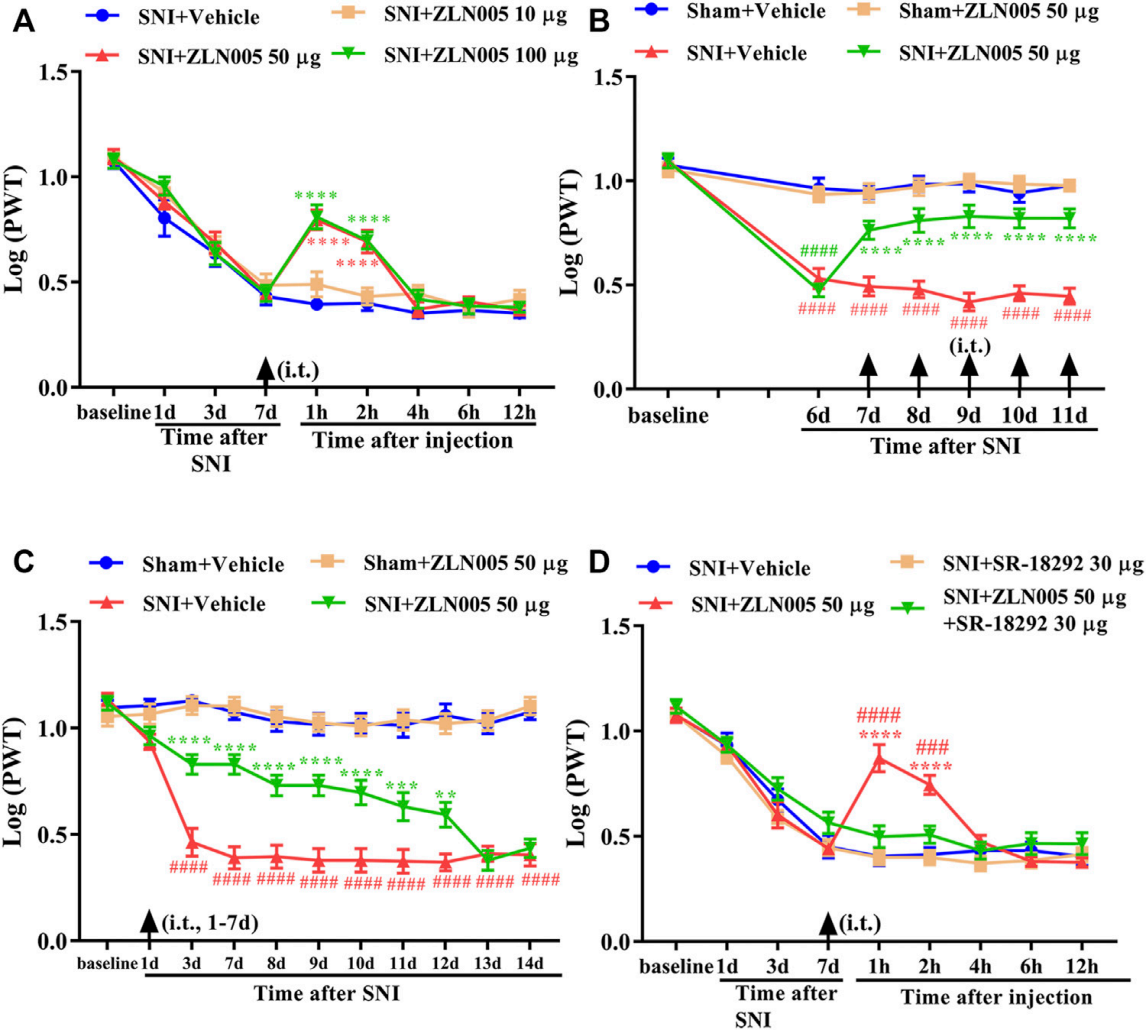
Analgesic effect of PGC-1α activator on mechanical allodynia in neuropathic pain rats. **(A)** A single dose of ZLN005 (50 and 100 μg) significantly increased the PWT in SNI rats, peaking at 1 h, and lasted for at least 2 h (^****^ *p* < 0.0001 compared with SNI + Vehicle group, n = 10 in each group). **(B)** Repetitive injections of ZLN005 (50 μg, i. t.) considerably reversed established mechanical allodynia in SNI rats (^****^ *p* < 0.0001 compared with SNI + Vehicle group, ^####^ *p* < 0.0001 compared with Sham + Vehicle group, n = 10 in each group). **(C)** The PWT was significantly increased from day 3 to day 12 in ZLN005-treated SNI rats compared with vehicle-treated SNI rats (^**^ *p* < 0.01, ^***^ *p* < 0.001, ^****^ *p* < 0.0001 compared with SNI + Vehicle group, ^####^ *p* < 0.0001 compared with Sham + Vehicle group, n = 10 in each group). **(D)** The analgesic effects of ZLN005 were reversed by PGC-1α inhibitor SR-18292 (^****^ *p* < 0.0001 compared with SNI + Vehicle group, ^###^ *p* < 0.001, ^####^ *p* < 0.0001 compared with SNI + ZLN005 50 μg + SR-1892 30-μg group, n = 10 in each group).

We then determined whether the activation of PGC-1α, the master regulator of mitochondrial biogenesis, could attenuate mechanical allodynia in rats with neuropathic pain. To determine whether a single dose of ZLN005 (PGC-1α activator) could attenuate established mechanical allodynia in rats with SNI, ZLN005 (10, 50, or 100 μg, i. t.) was administered on day 7 after surgery. Behavioral tests were conducted before ZLN005 injection and 1, 2, 4, 6, and 12 h after the injection. There was no significant change in PWT in SNI rats treated with 10 μg ZLN005 (Figure 2A, *p* > 0.05, compared with the SNI + Vehicle group, n = 10 in each group). However, ZLN005 (50 and 100 μg) significantly increased the PWT in SNI rats, peaking at 1 h, and lasting for at least 2 h (Figure 2A, ^****^ *p* < 0.0001 compared with SNI + Vehicle group, n = 10 in each group). To determine whether repeated injection of a PGC-1α activator could reverse mechanical allodynia in SNI rats, ZLN005 (50 μg, i. t.) was administered once daily for five consecutive days starting from day 7. Behavioral tests were performed on days 6 and 1 h after ZLN005 injection. As shown in Figure 2B, repetitive injections of ZLN005 (50 μg, i. t.) considerably reversed established mechanical allodynia in SNI rats (^****^ *p* < 0.0001 compared with SNI + Vehicle group, ^####^ *p* < 0.0001 compared with Sham + Vehicle group, n = 10 in each group). To determine whether early treatment with a PGC-1α activator could suppress the development of mechanical allodynia in SNI rats, ZLN005 (50 μg, i. t.) was administered once daily for seven consecutive days starting from day 1 after surgery. The behavioral test was conducted before surgery and on days 1, 3, 7, 8, 9, 10, 11, 12, 13, and 14 after surgery. As shown in Figure 2C, the PWT was significantly increased from day 3 to day 12 in ZLN005-treated SNI rats compared with vehicle-treated SNI rats (^**^ *p* < 0.01, ^***^ *p* < 0.001, ^****^ *p* < 0.0001 compared with SNI + Vehicle group, ^####^ *p* < 0.0001 compared with Sham + Vehicle group, n = 10 in each group). To determine whether a PGC-1α inhibitor could reverse the analgesic effects of ZLN005, SR-18292 (30 μg, i. t.) was administered 30 min before ZLN005. Behavioral tests were conducted before SR-18292 injection and 1, 2, 4, 6, and 12 h after ZLN005 injection. As shown in Figure 2D, the analgesic effects of ZLN005 were reversed by SR-18292 (PGC-1α inhibitor) (^****^ *p* < 0.0001 compared with the SNI + Vehicle group, ^###^ *p* < 0.001, ^####^ *p* < 0.0001 compared with the SNI + ZLN005 50 μg + SR-18292 30 μg group, n = 10 in each group). These results indicate that ZLN005 markedly attenuates established mechanical allodynia in SNI rats and delays the development of mechanical allodynia in SNI rats.

### Expression and Cellular Localization of 5-HT_1F_ Receptor in the Spinal Cord of Sham and Neuropathic Pain Rats

**FIGURE 3:**
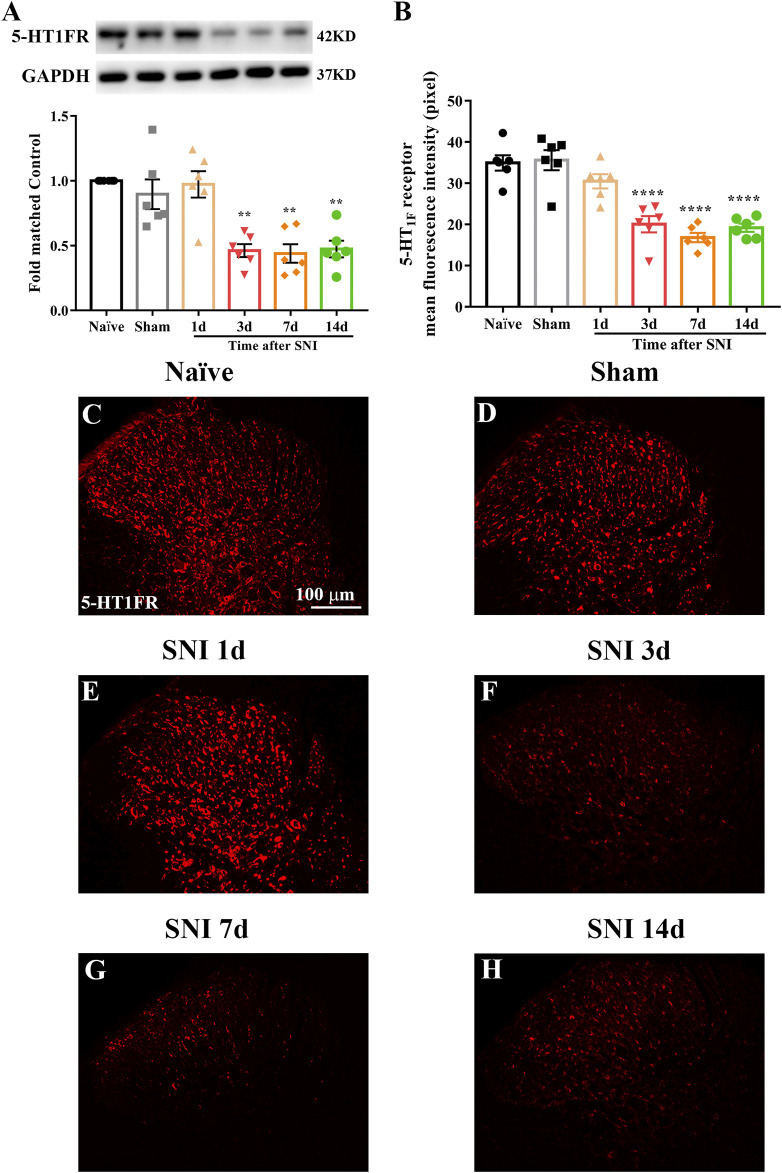
Expression of 5-HT_1F_ receptor in the spinal cord of sham and neuropathic pain rats. **(A)** The protein level of 5-HT_1F_ receptor was significantly decreased in SNI rats, beginning at day 3 after surgery and remain at low level until day 14 (^**^ *p* < 0.01 compared with sham group, n = 6 in each group). **(B)** Histogram showing the mean intensity of 5-HT1F receptor immunofluorescent activity was significantly decreased in SNI rats, beginning at day 3 after surgery and remain at low level until day 14 (****p < 0.0001 compared with sham group, n = 6 in each group). **(C–H)** Representative photomicrographs showed that neuropathic pain induced the downregulation of 5-HT_1F_ receptor in the spinal cord.

**FIGURE 4:**
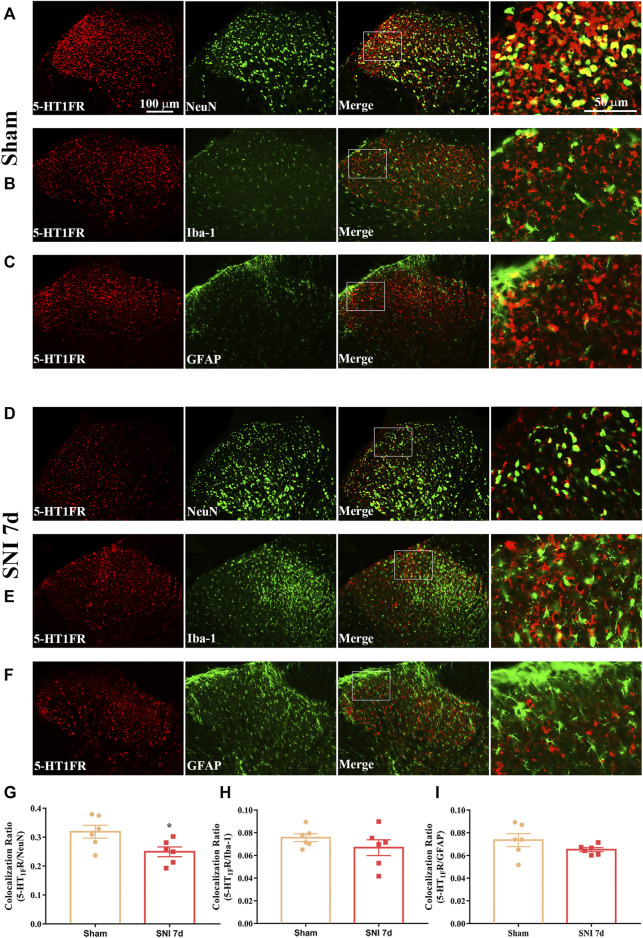
Double immunofluorescence of 5-HT_1F_ receptor and NeuN, GFAP, and Iba1 in the spinal cord of sham and neuropathic pain rats. **(A)** Double immunofluorescence of 5-HT_1F_ receptor and NeuN in the spinal cord of sham rats. **(B)** Double immunofluorescence of 5-HT_1F_ receptor and Iba-1 in the spinal cord of sham rats. **(C)** Double immunofluorescence of 5-HT_1F_ receptor and GFAP in the spinal cord of sham rats. **(D)** Double immunofluorescence of 5-HT_1F_ receptor and NeuN in the spinal cord of SNI rats. **(E)** Double immunofluorescence of 5-HT_1F_ receptor and Iba-1 in the spinal cord of SNI rats. **(F)** Double immunofluorescence of 5-HT_1F_ receptor and GFAP in the spinal cord of SNI rats. **(G–I)** Neuropathic pain induced decreased colocalization of 5-HT_1F_ receptor with neurons (NeuN), while the colocalization of 5-HT_1F_ receptor with astrocytes (GFAP), and microglia (Iba-1) remained sparse in the spinal cord (*p < 0.05 compared with sham group, n = 6 in each group).

**FIGURE 5:**
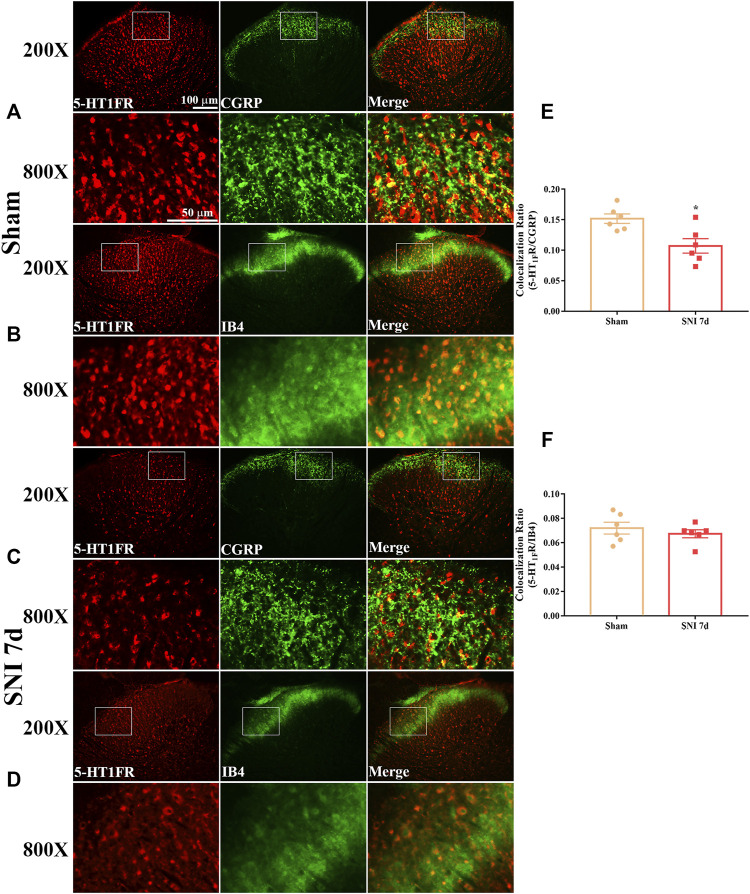
Double immunofluorescence of 5-HT_1F_ receptor and CGRP and IB4 in the spinal cord of sham and neuropathic pain rats. **(A)** Double immunofluorescence of 5-HT_1F_ receptor and CGRP in the spinal cord of sham rats. **(B)** Double immunofluorescence of 5-HT_1F_ receptor and IB4 in the spinal cord of sham rats. **(C)** Double immunofluorescence of 5-HT_1F_ receptor and CGRP in the spinal cord of SNI rats. **(D)** Double immunofluorescence of 5-HT_1F_ receptor and IB4 in the spinal cord of SNI rats. **(E,F)** Neuropathic pain induced decreased colocalization of 5-HT_1F_ receptor with CGRP, while the colocalization of 5-HT_1F_ receptor with IB4 was not affected by neuropathic pain in the spinal cord (*p < 0.05 compared with sham group, n = 6 in each group).

Western blotting and immunofluorescence were used to determine the expression and cellular localization of 5-HT_1F_ receptors in sham and neuropathic pain rats. As shown in Figure 3A, the expression of 5-HT_1F_ receptor was significantly decreased in SNI rats, beginning at day 3 after surgery and remaining at a low level until day 14 (^**^ *p* < 0.01, compared with sham group, n = 6 in each group). Consistently, the immunofluorescence results showed decreased expression of 5-HT_1F_ receptors in the spinal cord dorsal horn of SNI rats (Figures 3B–H, ^****^ *p* < 0.0001 compared with the sham group, n = 6 in each group). Our double immunofluorescence results showed that 5-HT_1F_ receptors were mainly colocalized with NeuN (a neuronal marker) in the spinal cord dorsal horn of sham and SNI rats, while the colocalization of 5-HT_1F_ receptors with NeuN was decreased in the SNI rats (Figure 4, *p < 0.05, compared with sham group, n = 6 in each group). Moreover, 5-HT_1F_ receptors were also colocalized with CGRP and IB4 in the spinal cord dorsal horn of sham and SNI rats, while the colocalization of 5-HT_1F_ receptors with CGRP was decreased in SNI rats (Figure 5, *p < 0.05, compared with sham group, n = 6 in each group).

### Analgesic Effect of 5-HT_1F_ Receptor Agonist on Mechanical Allodynia in Neuropathic Pain Rats

**FIGURE 6:**
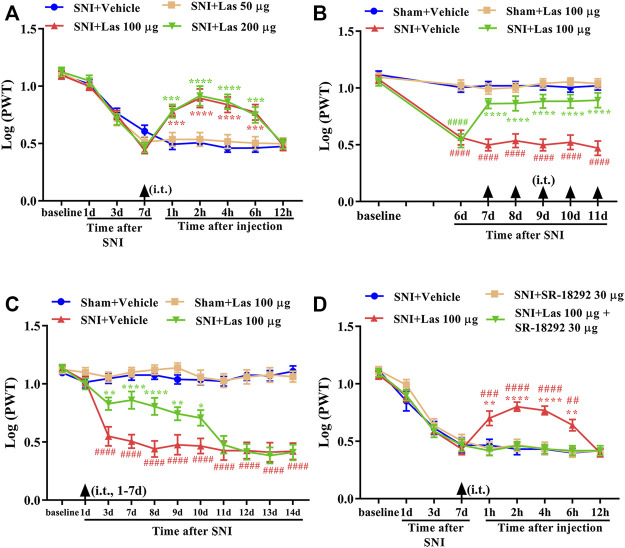
Analgesic effect of 5-HT_1F_ receptor agonist on mechanical allodynia in neuropathic pain rats. **(A)** A single dose of lasmiditan (100 and 200 μg) markedly increased the PWT in SNI rats, beginning at 1 h, peaking at 2 h, and lasted for at least 6 h (Figure 7A, ^***^ *p* < 0.001, ^****^ *p* < 0.0001 compared with SNI + Vehicle group, n = 10 in each group). **(B)** Repetitive injections of lasmiditan (100 μg, i. t.) considerably reversed established mechanical allodynia in SNI rats (^****^ *p* < 0.0001 compared with SNI + Vehicle group, ^####^ *p* < 0.0001 compared with Sham + Vehicle group, n = 10 in each group). **(C)** The PWT was significantly increased from day 3 to day 10 in lasmiditan-treated SNI rats compared with vehicle-treated SNI rats (^*^ *p* < 0.05, ^**^ *p* < 0.01, ^****^ *p* < 0.0001 compared with SNI + Vehicle group, ^####^ *p* < 0.0001 compared with Sham + Vehicle group, n = 10 in each group). **(D)** The analgesic effect of lasmiditan was entirely reversed by PGC-1α inhibitor SR-18292 (^**^ *p* < 0.01, ^****^ *p* < 0.0001 compared with SNI + Vehicle group, ^##^ *p* < 0.01, ^###^ *p* < 0.001, ^####^ *p* < 0.0001 compared with SNI + Las 100 μg + SR-1892 30 μg group, n = 10 in each group).

To determine whether a single dose of lasmiditan (5-HT_1F_ receptor agonist) could attenuate established mechanical allodynia in SNI rats, lasmiditan (50, 100, or 200 μg, i. t.) was administered on day 7 after surgery. Behavioral tests were conducted before lasmiditan injection and 1, 2, 4, 6, and 12 h after the injection. There was no significant change in PWT in SNI rats treated with 50-μg lasmiditan (Figure 6A, *p* > 0.05, compared with SNI + Vehicle group, n = 10 in each group). However, lasmiditan (100 and 200 μg) markedly increased the PWT in SNI rats, beginning at 1 h, peaking at 2 h, and lasting for at least 6 h (Figure 6A, ^***^ *p* < 0.001, ^****^ *p* < 0.0001 compared with SNI + Vehicle group, n = 10 in each group). To determine whether repeated injection of a 5-HT_1F_ receptor agonist could reverse mechanical allodynia in SNI rats, lasmiditan (100 μg, i. t.) was administered once daily for five consecutive days starting from day 7. The behavioral test was performed on day 6 and 2 h after lasmiditan injection. As shown in Figure 6B, repetitive injections of lasmiditan (100 μg, i. t.) considerably reversed established mechanical allodynia in SNI rats (^****^ *p* < 0.0001 compared with SNI + Vehicle group, ^####^ *p* < 0.0001 compared with Sham + Vehicle group, n = 10 in each group). To determine whether early treatment with a 5-HT_1F_ receptor agonist can suppress the development of mechanical allodynia in SNI rats, lasmiditan (100 μg, i. t.) was administered once daily for seven consecutive days starting from day 1 after the surgery. The behavioral test was conducted before surgery and on days 1, 3, 7, 8, 9, 10, 11, 12, 13, and 14 after surgery. As shown in Figure 6C, the PWT was significantly increased from day 3 to day 10 in lasmiditan-treated SNI rats compared with vehicle-treated SNI rats (^*^ *p* < 0.05, ^**^ *p* < 0.01, ^****^ *p* < 0.0001 compared with SNI + Vehicle group, ^####^ *p* < 0.0001 compared with Sham + Vehicle group, n = 10 in each group). To determine whether a PGC-1α inhibitor could reverse the analgesic effects of lasmiditan, SR-18292 (30 μg, i. t.) was administered 30 min before lasmiditan. Behavioral tests were conducted before SR-18292 injection and 1, 2, 4, 6, and 12 h after lasmiditan injection. As shown in Figure 6D, the analgesic effects of lasmiditan were completely reversed by the PGC-1α inhibitor SR-18292 (^*^ *p* < 0.05, ^**^ *p* < 0.01, ^****^ *p* < 0.0001 compared with the SNI + Vehicle group, ^##^ *p* < 0.01, ^###^ *p* < 0.001, ^####^ *p* < 0.0001 compared with SNI + Las 100 μg + SR-1892 30 μg group, n = 10 in each group). These results indicate that lasmiditan can significantly attenuate established mechanical allodynia in SNI rats and delay the development of mechanical allodynia in SNI rats. Moreover, activation of PGC-1α contributes to the analgesic effects of lasmiditan.

### Effect of 5-HT_1F_ Receptor Agonist on PWT and Mitochondrial Biogenesis in Naïve Rats

**FIGURE 7:**
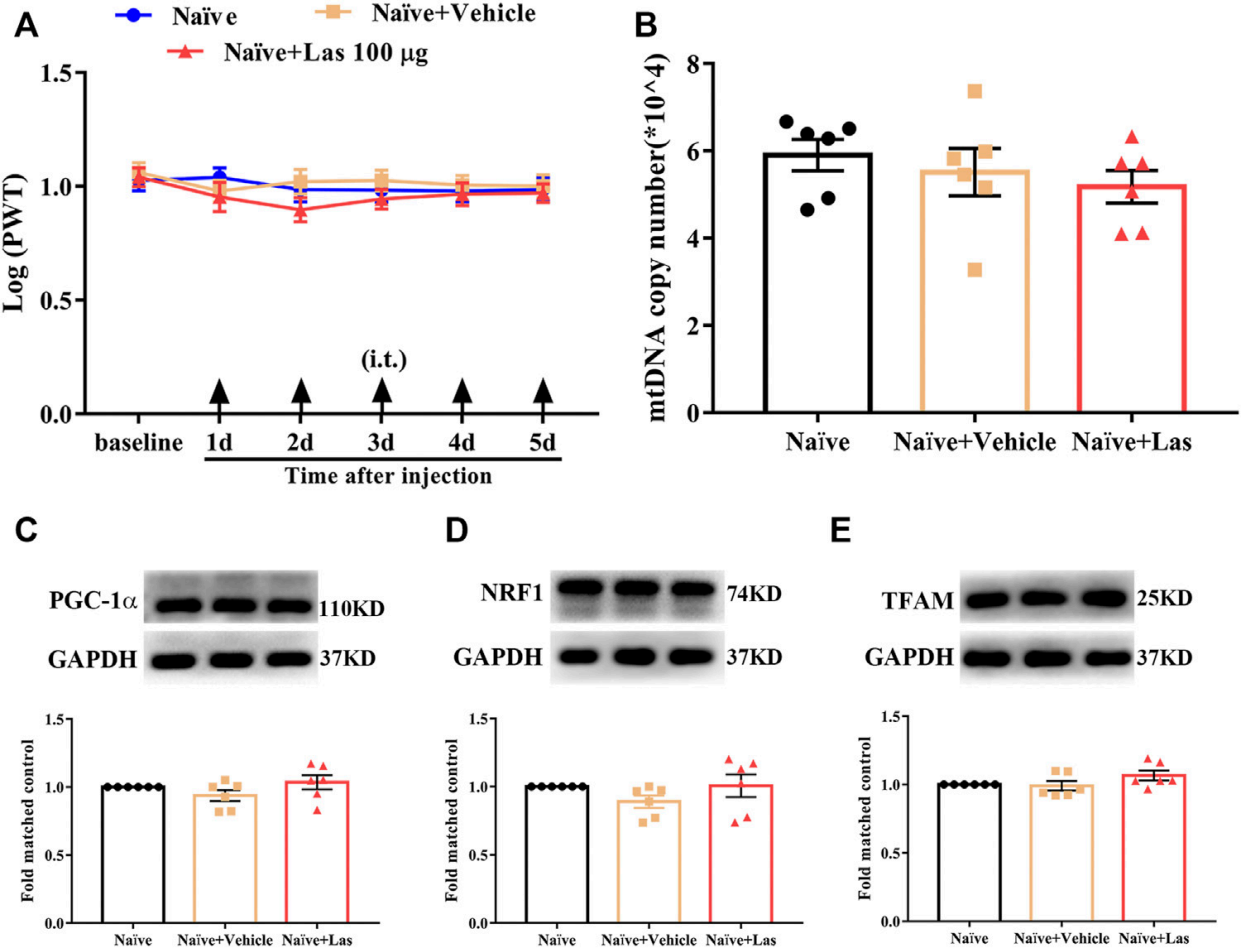
Effect of 5-HT_1F_ receptor agonist on PWT and mitochondrial biogenesis in naïve rats. **(A)** No significant change regarding PWT was observed after lasmiditan injection at any time of the observation period (*p* > 0.05 compared with Naïve + Vehicle group, n = 10 in each group). **(B)** No significant change regarding mtDNA copy number was observed after lasmiditan treatment (*p* > 0.05 compared with Naïve + Vehicle group, n = 6 in each group). **(C)** No significant change regarding the protein level of PGC-1α was observed after lasmiditan treatment (*p* > 0.05 compared with Naïve + Vehicle group, n = 6 in each group). **(D)** No significant change regarding the protein level of NRF1 was observed after lasmiditan treatment (*p* > 0.05 compared with Naïve + Vehicle group, n = 6 in each group). **(E)** No significant change regarding the protein level of TFAM was observed after lasmiditan treatment (*p* > 0.05 compared with Naïve + Vehicle group, n = 6 in each group).

To determine whether the 5-HT_1F_ receptor agonist affected mtDNA copy number and PWT in naïve rats, lasmiditan (100 μg, i. t.) was administered once daily for five consecutive days. The behavioral test was performed 2 h after the lasmiditan injection each day. As shown in Figures 7A,B, no significant change in the PWT and mtDNA copy number was observed after lasmiditan injection (*p* > 0.05, compared with Naïve + Vehicle group, n = 10 in each group). To determine whether the 5-HT_1F_ receptor agonist affected mitochondrial biogenesis in naïve rats, lasmiditan (100 μg, i. t.) was administered once daily for five consecutive days. Two hours after the final administration, the L4–6 spinal cord was collected to examine protein level of PGC-1α, NRF1, and TFAM. As shown in Figures 7C–E, no significant changes in protein levels of PGC-1α, NRF1, and TFAM were observed after lasmiditan treatment (*p* > 0.05, compared with the Naïve + Vehicle group, n = 6 in each group). These results suggest that lasmiditan treatment in the current protocol has no impact on PWT or mitochondrial biogenesis in naïve rats.

### Effect of 5-HT_1F_ Receptor Agonist on Mitochondrial Biogenesis in the Spinal Cord of Neuropathic Pain Rats

**FIGURE 8:**
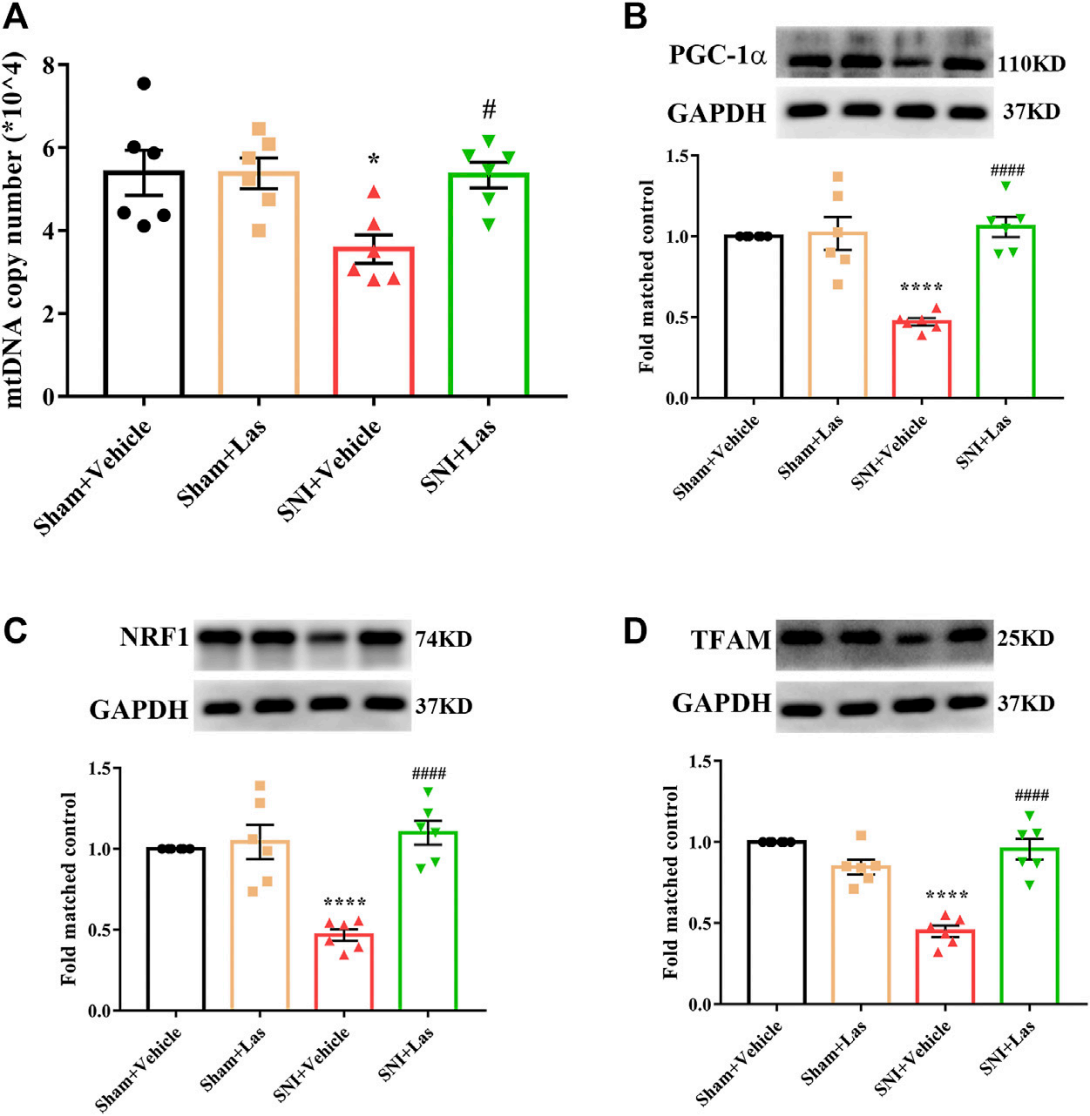
Effect of 5-HT_1F_ receptor agonist on mitochondrial biogenesis in the spinal cord of neuropathic pain rats. **(A)** To determine whether 5-HT_1F_ receptor agonist affect mitochondrial biogenesis in SNI rats, lasmiditan (100 μg, i. t.) was given once daily for five consecutive days starting from day 7. Two hours after the final administration, the L4–6 spinal cord was collected. The mtDNA copy number was significantly downregulated in SNI rats, which was reversed by Lasmiditan treatment (^*^ *p* < 0.05, compared with Sham + Vehicle group, ^#^ *p* < 0.05 compared with SNI + Vehicle group, n = 6 in each group). **(B)** The protein level of PGC-1α was significantly downregulated in SNI rats, which was reversed by lasmiditan treatment (^****^ *p* < 0.0001 compared with Sham + Vehicle group, ^####^ *p* < 0.0001 compared with SNI + Vehicle group, n = 6 in each group). **(C)** The protein level of NRF1 was significantly downregulated in SNI rats, which was reversed by lasmiditan treatment (^****^ *p* < 0.0001 compared with Sham + Vehicle group, ^####^ *p* < 0.0001 compared with SNI + Vehicle group, n = 6 in each group). **(D)** The protein level of TFAM was significantly downregulated in SNI rats, which was reversed by lasmiditan treatment (^****^ *p* < 0.0001 compared with Sham + Vehicle group, ^####^ *p* < 0.0001 compared with SNI + Vehicle group, n = 6 in each group).

To determine whether the 5-HT_1F_ receptor agonist affected mitochondrial biogenesis in SNI rats, lasmiditan (100 μg, i. t.) was administered once daily for five consecutive days starting from day 7. Two hours after the final administration, the L4–6 spinal cord was collected. As shown in Figure 8, the mtDNA copy number and the protein levels of PGC-1α, NRF1, and TFAM were significantly downregulated in SNI rats, which was reversed by lasmiditan treatment (^*^ *p* < 0.05, ^****^ *p* < 0.0001 compared with the Sham + Vehicle group, ^#^ *p* < 0.05, ^####^ *p* < 0.0001 compared with the SNI + Vehicle group, n = 6 in each group). These results suggest that lasmiditan restored mitochondrial biogenesis in the spinal cord of rats with SNI.

### Effect of 5-HT_1F_ Receptor Agonist on Neuroinflammation in the Spinal Cord of Neuropathic Pain Rats

**FIGURE 9:**
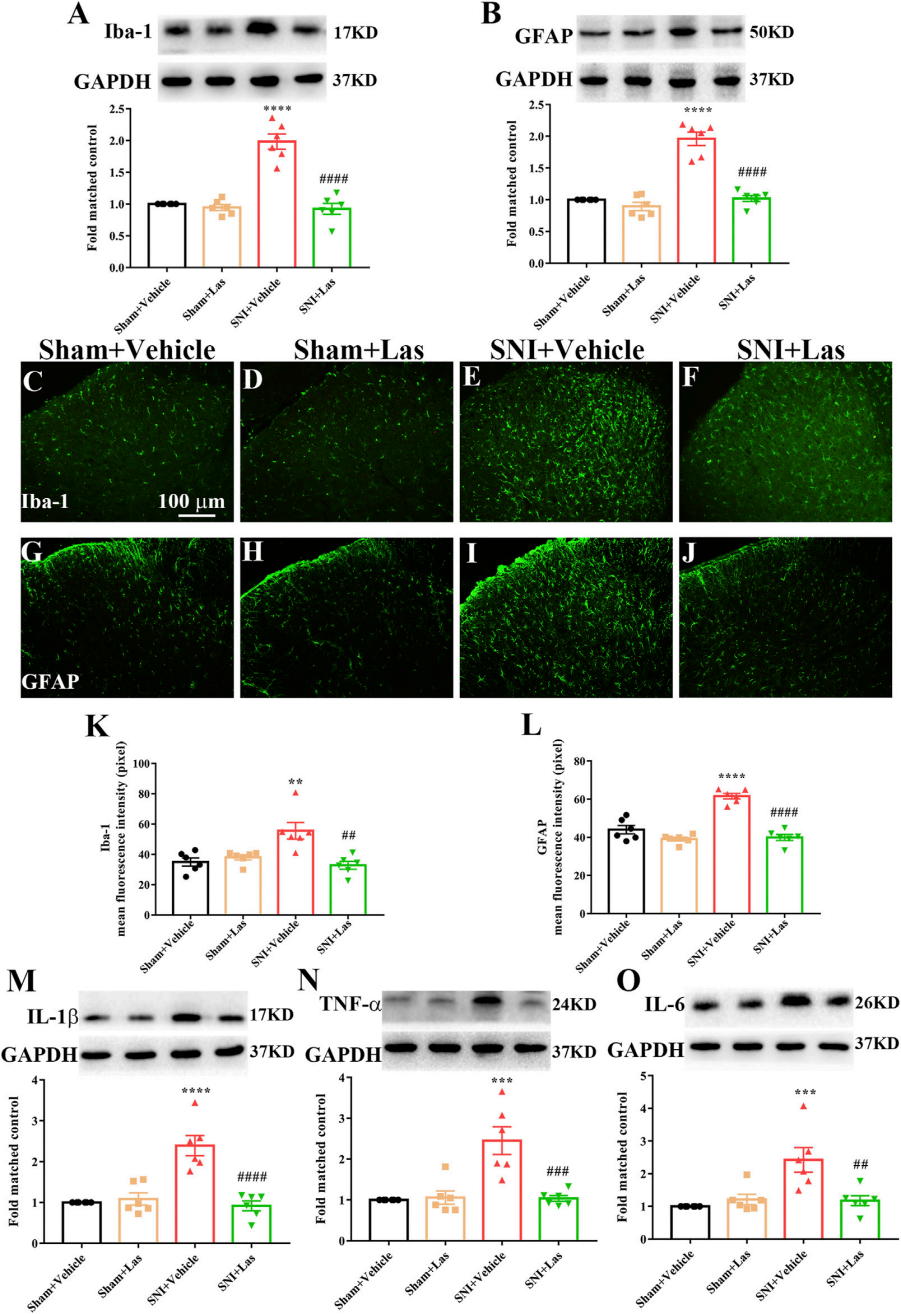
Effect of 5-HT_1F_ receptor agonist on neuroinflammation in the spinal cord of neuropathic pain rats. **(A,B)** To determine whether 5-HT_1F_ receptor agonist affected neuroinflammation in SNI rats, lasmiditan (100 μg, i. t.) was given once daily for five consecutive days starting from day 7. Two hours after the final administration, the L4–6 spinal cord was collected. The protein level of Iba1 and GFAP were significantly increased in the spinal cord of SNI rats, which were markedly suppressed by lasmiditan treatment (^***^ *p* < 0.001, ^****^ *p* < 0.0001 compared with Sham + Vehicle group, ^##^ *p* < 0.01, ^###^ *p* < 0.001, ^####^ *p* < 0.0001 compared with SNI + Vehicle group, n = 6 in each group). **(C–L)** The immunofluorescence results were consistent with the western blot (^***^ *p* < 0.001, ^****^ *p* < 0.0001 compared with Sham + Vehicle group, ^##^ *p* < 0.01, ^###^ *p* < 0.001, ^####^ *p* < 0.0001 compared with SNI + Vehicle group, n = 6 in each group). **(M–O)** Lasmiditan treatment also inhibited the upregulation of proinflammatory cytokines including IL-1β, IL-6, and TNF-α (Figure 10M-O, ^*^ *p* < 0.05, ^****^ *p* < 0.0001 compared with Sham + Vehicle group, ^#^ *p* < 0.05, ^####^ *p* < 0.0001 compared with SNI + Vehicle group, n = 6 in each group).

To determine whether the 5-HT_1F_ receptor agonist affected neuroinflammation in SNI rats, lasmiditan (100 μg, i. t.) was administered once daily for five consecutive days starting from day 7. Two hours after the final administration, the L4–6 spinal cord was collected. As shown in Figure 9A-L, the expression of Iba1 and GFAP was significantly increased in the spinal cord of SNI rats, which was markedly suppressed by lasmiditan treatment (^**^ *p* < 0.01, ^****^ *p* < 0.0001 compared with the Sham + Vehicle group, ^##^ *p* < 0.01, ^####^ *p* < 0.0001 compared with the SNI + Vehicle group, n = 6 in each group). Lasmiditan treatment also inhibited the upregulation of pro-inflammatory cytokines, including IL-1β, IL-6, and TNF-α (Figure 9M-O, ^*^ *p* < 0.05, ^****^ *p* < 0.0001 compared with Sham + Vehicle group, ^#^ *p* < 0.05, ^####^ *p* < 0.0001 compared with the SNI + Vehicle group, n = 6 in each group). These results suggest that lasmiditan suppressed neuroinflammation in the spinal cord of rats with SNI.

## Discussion

**FIGURE 10:**
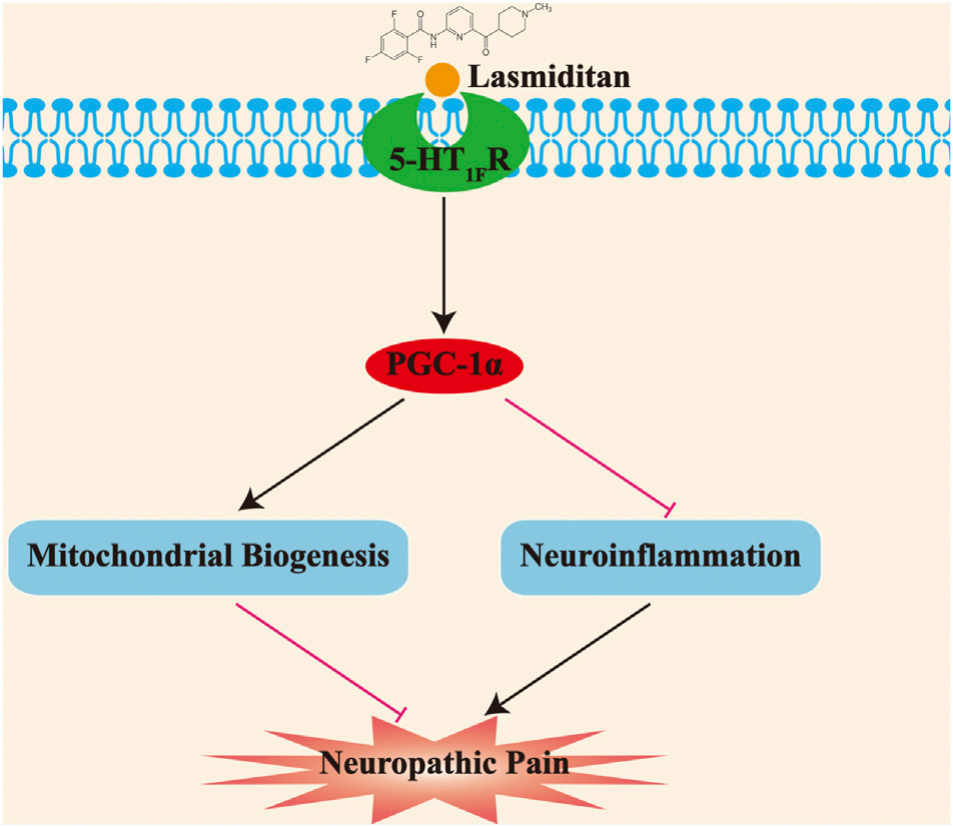
Schematic diagram demonstrating that activation of 5-HT_1F_ receptor improves mechanical allodynia induced by neuropathic pain. The impaired mitochondrial biogenesis promotes the development of neuropathic pain; moreover, activation of 5-HT_1F_ receptor with lasmiditan ameliorates mechanical allodynia induced by neuropathic pain through prompting mitochondrial biogenesis and suppressing neuroinflammation.

In this study, we showed that 1) mitochondrial biogenesis was impaired in the spinal cord of SNI rats; 2) activation of PGC-1α, the master regulator of mitochondrial biogenesis, attenuated established mechanical allodynia, and delayed the onset of mechanical allodynia in SNI rats; 3) neuronal 5-HT_1F_ receptor was significantly downregulated in the spinal cord of rats with neuropathic pain; 4) lasmiditan attenuated established mechanical allodynia and delayed the onset of mechanical allodynia in SNI rats; 5) lasmiditan treatment restored mitochondrial biogenesis in the spinal cord of SNI rats; and 6) lasmiditan treatment attenuated neuroinflammation in the spinal cord of SNI rats. Taken together, these results indicate that the 5-HT_1F_ receptor agonist lasmiditan ameliorates mechanical allodynia in neuropathic pain via induction of mitochondrial biogenesis and suppression of neuroinflammation in the spinal cord (Figure 10).

The excessive accumulation of reactive oxygen species (ROS) leads to oxidative stress. Our previous study and others have demonstrated the potent analgesic effects of ROS scavengers against cancer-induced bone pain and neuropathic pain (Kim H. K. et al., 2010; Zhou et al., 2018). Nuclear factor erythroid-2-related factor 2 (Nrf2) is a key regulator of endogenous antioxidant defense. Previously, we and others have shown that Nrf2 activators significantly alleviated mechanical allodynia in neuropathic pain (Kim D. et al., 2010; Zhou et al., 2020a). These results indicated that oxidative stress plays a vital role in the development of neuropathic pain. Oxidative phosphorylation in mitochondria is one of the main sources of ROS (Martin-Montanez et al., 2019). Notably, oxidative stress disrupts the mitochondrial respiratory chain, disturbs Ca^2+^ homeostasis, and increases mitochondrial membrane permeability (Elfawy and Das 2019). Therefore, oxidative stress may result in mitochondrial dysfunction and vice versa. Mitochondrial dysfunction has emerged as a key contributor to the development of neuropathic pain (Areti et al., 2016; Dai et al., 2020). Impaired mitochondrial biogenesis has been reported to contribute to mitochondrial dysfunction in neurological diseases such as Alzheimer’s disease and spinal cord injury (Sheng et al., 2012; Scholpa et al., 2019). In this study, we found that mtDNA copy number and protein level of PGC-1α, NRF1, and TFAM were significantly downregulated in the spinal cord of SNI rats, indicating impaired mitochondrial biogenesis. However, it is noteworthy that the mtDNA copy number progressively decreased following SNI, whereas the protein level of PGC-1α was relatively stable. This discrepancy might be due to the fact that mitochondrial biogenesis is also regulated by other genes except PGC-1α. PGC-1α is a master regulator of mitochondrial biogenesis. Therefore, we hypothesized that activation of PGC-1α might attenuate neuropathic pain. Our results showed that a single dose of ZLN005 (a PGC-1α activator) attenuated established mechanical allodynia in rats with SNI, which was blocked by the PGC-1α inhibitor SR-18292. These results indicate that the activation of PGC-1α contributes to the analgesic effects of ZLN005. In addition, repetitive injections of ZLN005 considerably reversed established mechanical allodynia in SNI rats. Moreover, early treatment with ZLN005 for seven consecutive days from day 1 after surgery delayed the onset of mechanical allodynia in SNI rats. A recent study has reported that PGC-1α haploinsufficiency promotes pain chronification after burn injury (Miao et al., 2020). Moreover, a recent study demonstrated that activation of spinal PGC-1α suppressed morphine tolerance by reducing mitochondrial superoxide levels (Kashiwagi et al., 2021), indicating ameliorative effects of PGC-1α in oxidative stress. Given the pivotal role of oxidative stress in neurological diseases (Patel, 2016), it is plausible that PGC-1α activation attenuates neurological diseases through similar mechanisms. Collectively, our results demonstrate that impaired mitochondrial biogenesis might contribute to the development and maintenance of mechanical allodynia in rats with SNI.

Serotonin, in the central nervous system, has long been considered to play a vital role in modulating pain processing (Bardin, 2011). Accumulating evidence indicates a fundamental role of individual classes of 5-HT receptors in neuropathic pain (Wang et al., 2020). For example, intrathecal injection of 5-HT_1A_ and 5-HT_7_ receptor agonists showed potent analgesic effects (Lin et al., 2015; Newman-Tancredi et al., 2018). The 5-HT_1F_ receptor is a G-protein-coupled receptor subtype whose functions are not fully characterized. Lasmiditan, a highly selective 5-HT_1F_ receptor agonist, has been approved by the United States Food and Drug Administration for acute treatment of migraine (Lamb, 2019). Several lines of evidence have indicated that 5-HT_1F_ receptor agonists are potent inducers of mitochondrial biogenesis (Rasbach et al., 2010; Garrett et al., 2014; Scholpa et al., 2018; Dupre et al., 2019). However, the role of the 5-HT_1F_ receptors in neuropathic pain remains unclear. In this study, we examined the expression and cellular localization of 5-HT_1F_ receptors in the spinal cord. We found that the expression of 5-HT_1F_ receptors was significantly decreased in rats with SNI. Furthermore, our double immunofluorescence results showed that 5-HT_1F_ receptors were mainly colocalized with neurons in the dorsal horn of the spinal cord of sham and SNI rats. Our behavioral tests showed that a single dose of lasmiditan attenuated established mechanical allodynia in SNI rats, which was blocked by the PGC-1α inhibitor SR-18292. These results indicate that activation of PGC-1α contributes to the analgesic effects of lasmiditan. Moreover, repetitive injections of lasmiditan considerably reversed the established mechanical allodynia in rats with SNI. Furthermore, early treatment with lasmiditan for 7 consecutive days from day 1 after surgery delayed the onset of mechanical allodynia in SNI rats. A study has indicated that the half-life of ZLN005 is 11.46 ± 0.67 min in rats (Sun et al., 2018). A clinical study indicated that plasma concentrations of lasmiditan peaked at 1.5–2 h post dose, with a terminal half-life of approximately 4 h (Tsai et al., 2021). The biological half-life of ZLN005 was shorter than that of lasmiditan, which might explain the shorter analgesic effect of a single dose of ZLN005. About the specificity, lasmiditan could bind 5-HT_1F_ receptor, however, lasmiditan could also bind 5-HT_1B_ receptor (Hou et al., 2020). Although lasmiditan administration leads to increased expression of PGC-1α, it has an indirect effect. However, ZLN005 specifically upregulates the expression level of PGC-1α (Zhang et al., 2013). This may explain the shorter analgesic effect of lasmiditan when administered consecutively.

Notably, repetitive injections of lasmiditan had no significant effect on PWT in naïve rats. These results indicate that 5-HT_1F_ receptors are downregulated in the spinal cord of SNI rats, and lasmiditan possesses potent analgesic effects against SNI-induced neuropathic pain. Importantly, lasmiditan treatment had no significant effect on mitochondrial biogenesis in the spinal cord of naïve rats, but it restored mitochondrial biogenesis in the spinal cord of SNI rats. Recently, Simmons et al. found that naïve mice treated with the 5HT_1F_ receptor agonist LY344864 (2.0 mg/kg, i. p.) daily for 21 days displayed a 1.4-fold increase in mtDNA content and 1.3-fold increase in PGC-1α mRNA expression (Simmons et al., 2020). This discrepancy might be explained by differences in the drug, route of drug administration, treatment protocol, and animal species. Nevertheless, they found that LY344864 promoted recovery from spinal cord injury by inducing mitochondrial biogenesis. These results indicate that 5-HT_1F_ receptor agonists attenuate neuropathic pain by restoring mitochondrial biogenesis in the spinal cord. Lasmiditan is orally bioavailable and penetrates the central nervous system, with minimal off-target adverse effects (Lamb, 2019). Systemic administration of lasmiditan may have therapeutic potential for neuropathic pain.

Recent evidence indicates that PGC-1α activation exerts neuroprotective effects by suppressing neuroinflammation in animal models of Parkinson’s disease, depression, and brain injury (Corona and Duchen 2015; Fu et al., 2020; Han et al., 2021). Accumulating evidence from our laboratory and others has demonstrated the pivotal role of neuroinflammation in chronic pain (Chen et al., 2019; Jiang et al., 2020). Therefore, we tested the hypothesis that 5-HT_1F_ receptor agonists could attenuate neuropathic pain via suppression of neuroinflammation. As expected, repeated treatment with lasmiditan significantly inhibited the activation of microglia and astrocytes in the spinal cord. Lasmiditan also suppressed the upregulation of proinflammatory cytokines, including IL-1β, IL-6, and TNF-α. Although 5-HT_1F_ receptors are not expressed on microglia and astrocytes, the application of the 5-HT1F receptor agonist lasmiditan decreased the expression of Iba-1 and GFAP, as well as the glia-related inflammatory factor. This phenomenon might be explained by crosstalk between neurons and glial cells. A previous study demonstrated that peripheral nerve injury can rapidly induce the release of colony stimulating factor 1 (CSF1) in dorsal root ganglion neurons (Inoue and Tsuda, 2018). It is known that this increase results from the actions of interleukin-1β released from the surrounding satellite glia and its activating neural nuclear factor-κB (NF-κB) (Lim et al., 2017). CSF1 then acts on the CSF1 receptor, which is expressed on microglia and induces microgliosis (Inoue and Tsuda, 2018). Moreover, it has been proved that PGC-1α repressed the transcriptional activity of NF-κB (Eisele et al., 2013; Eisele et al., 2015). Furthermore, our study found that intrathecal injection with lasmiditan (5-HF_1F_ receptor agonist) could activate PGC-1α. Several recent studies have demonstrated that PGC-1α activation inhibits neuroinflammation (Yang et al., 2017; Han et al., 2021; Xie et al., 2021). Therefore, it is plausible that the application of the 5-HT_1F_ receptor agonist lasmiditan may inhibit neuroinflammation in the spinal cord via the activation of PGC-1α. These results indicate that 5-HT_1F_ receptor agonists attenuate neuropathic pain by suppressing neuroinflammation in the spinal cord. Notably, it was reported that lasmiditan could potentially block the release of CGRP and the neurotransmitter glutamate, thus preventing and possibly reversing the development of central sensitization (Clemow et al., 2020). Therefore, lasmiditan might attenuate neuropathic pain by inhibiting the release of CGRP from primary afferents. Moreover, it has been demonstrated that 5-HT_1F_ receptor is expressed in rat dorsal root ganglia neurons (Classey et al., 2010), and activation of peripheral 5-HT_1F_ receptor has analgesic effects (Granados-Soto et al., 2010), which suggests that a systemic route of administration with lasmiditan may have greater efficacy and clinical relevance.

In summary, this study provides the first evidence that impaired mitochondrial biogenesis in the spinal cord contributes to the development and maintenance of mechanical allodynia in rats with SNI. Moreover, lasmiditan significantly mitigated established neuropathic pain in SNI rats by inducing mitochondrial biogenesis and suppressing neuroinflammation. Inducers of mitochondrial biogenesis may be an encouraging therapeutic option for the management of neuropathic pain.

## Data Availability

The original contributions presented in the study are included in the article/Supplementary Material, further inquiries can be directed to the corresponding author.
