# Supplementary figures and images for: 5-HT1F Receptor Agonist Ameliorates Mechanical Allodynia in Neuropathic Pain via Induction of Mitochondrial Biogenesis and Suppression of Neuroinflammation
Source: Front Pharmacol. 2022 Mar 3;13:834570. doi: 10.3389/fphar.2022.834570 (PMC8927783; doi:10.3389/fphar.2022.834570)

**Fig 9A**

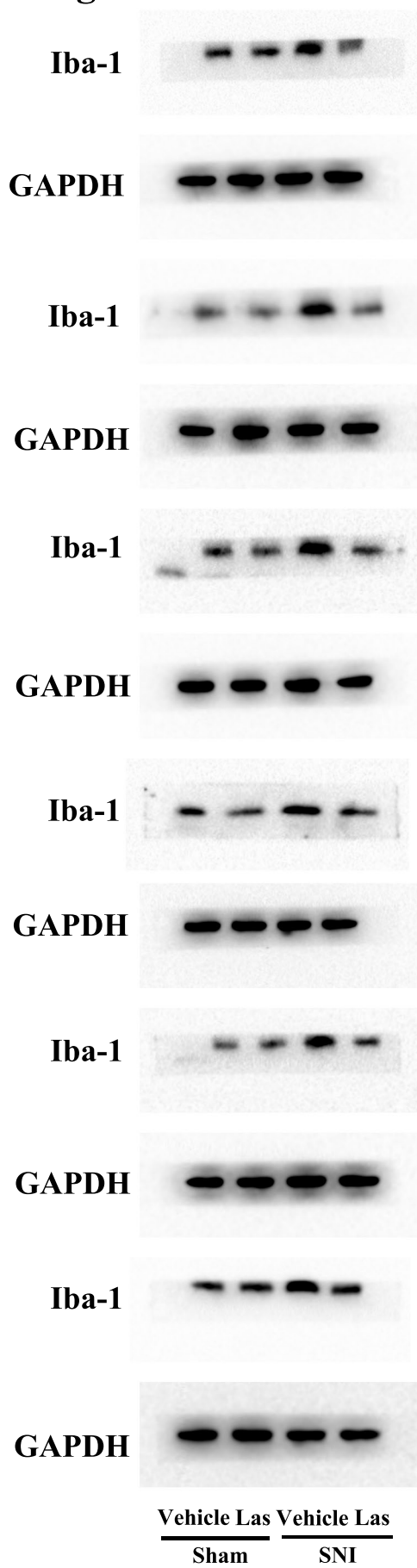

**Fig 9B**

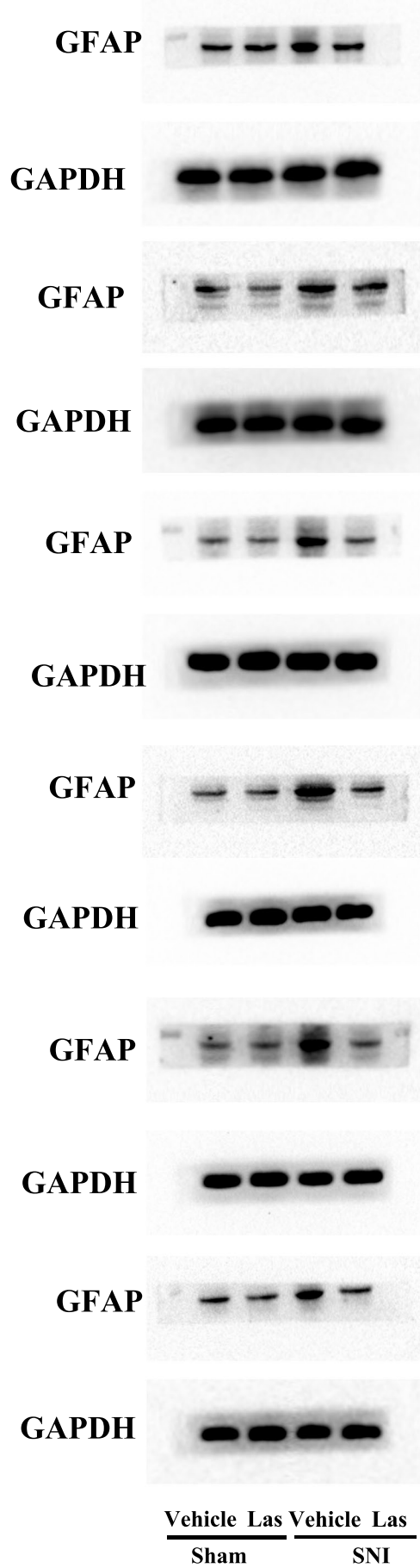

**Fig 9M**

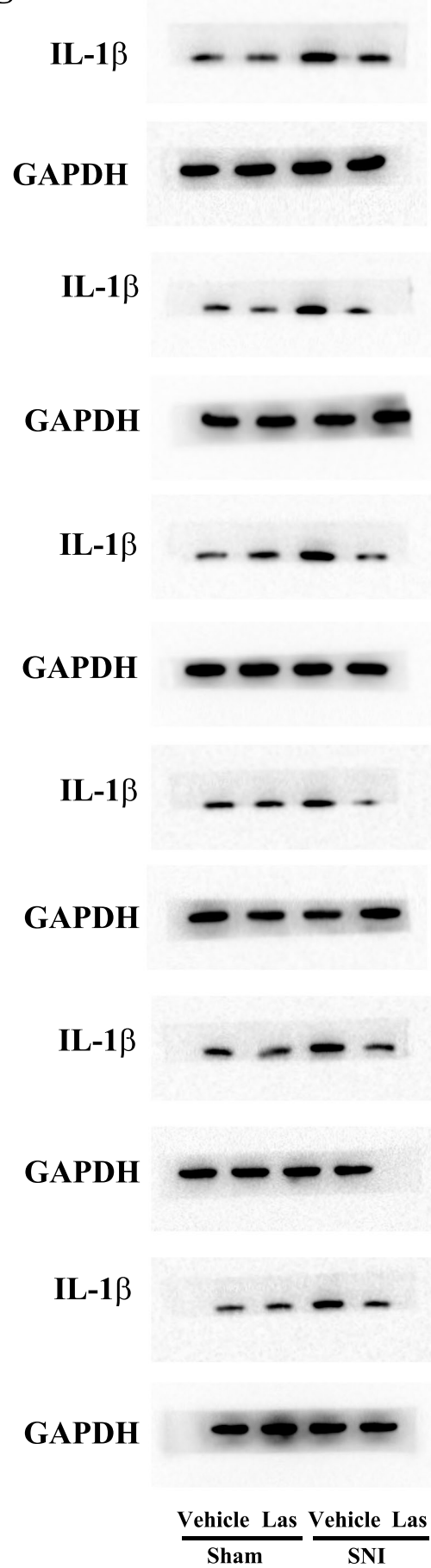

Supplement: Supplementary file 1 [file DataSheet1.ZIP › original blots/Fig 9A-B,M.pdf]

**Fig 1C**

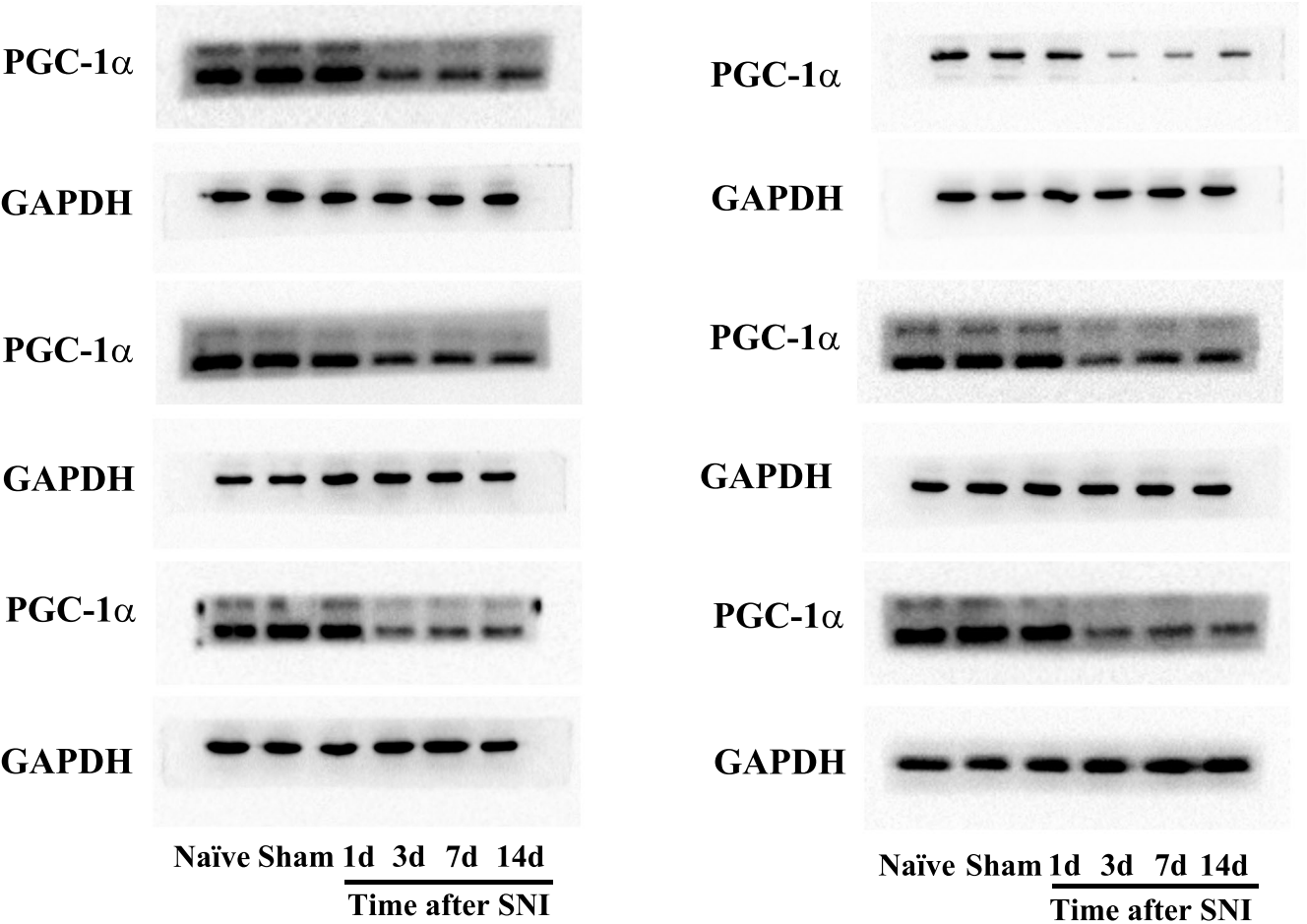

**Fig 1D**

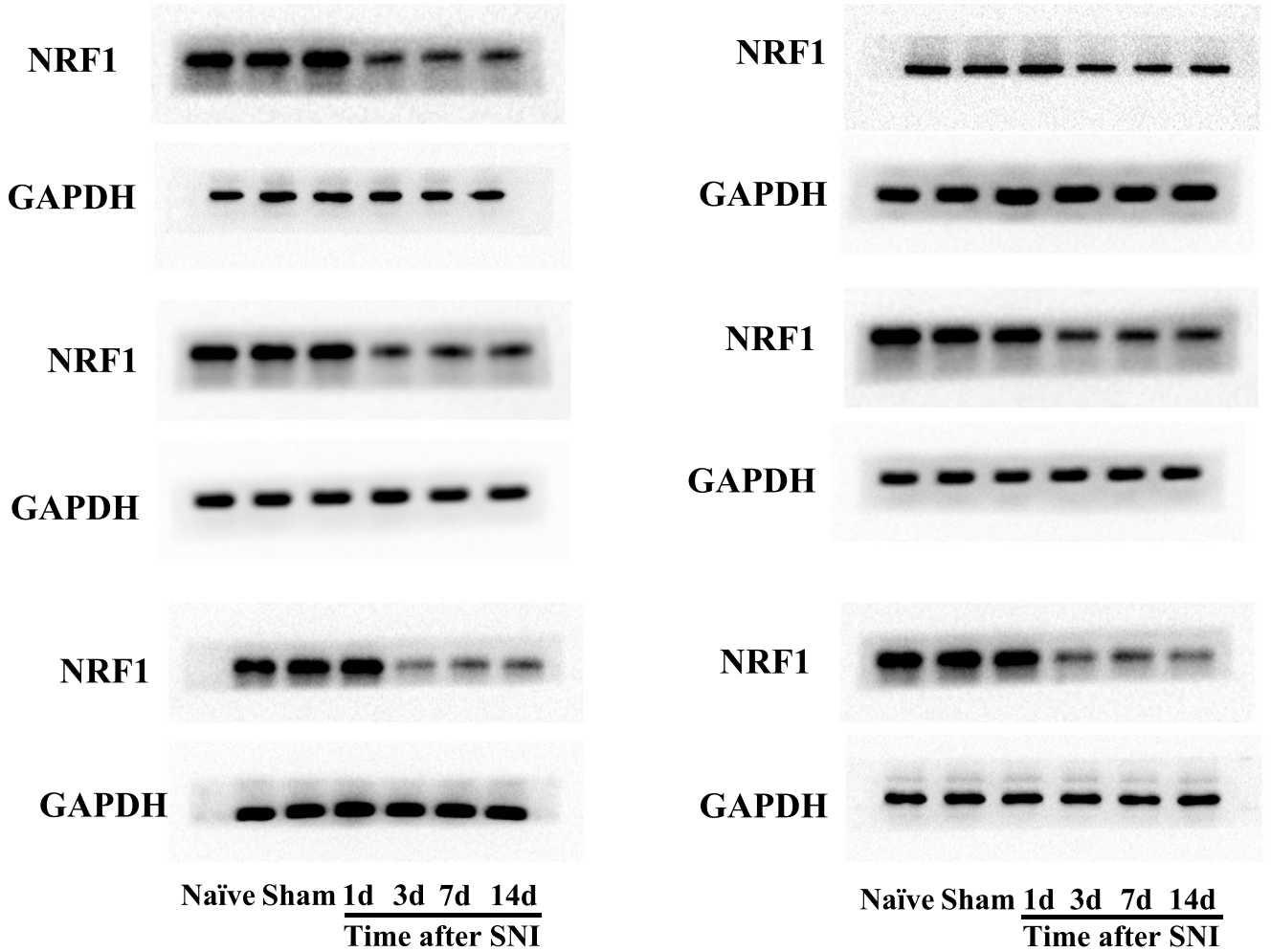

Supplement: Supplementary file 1 [file DataSheet1.ZIP › original blots/Fig. 1C-D.pdf]

**Fig 1E**

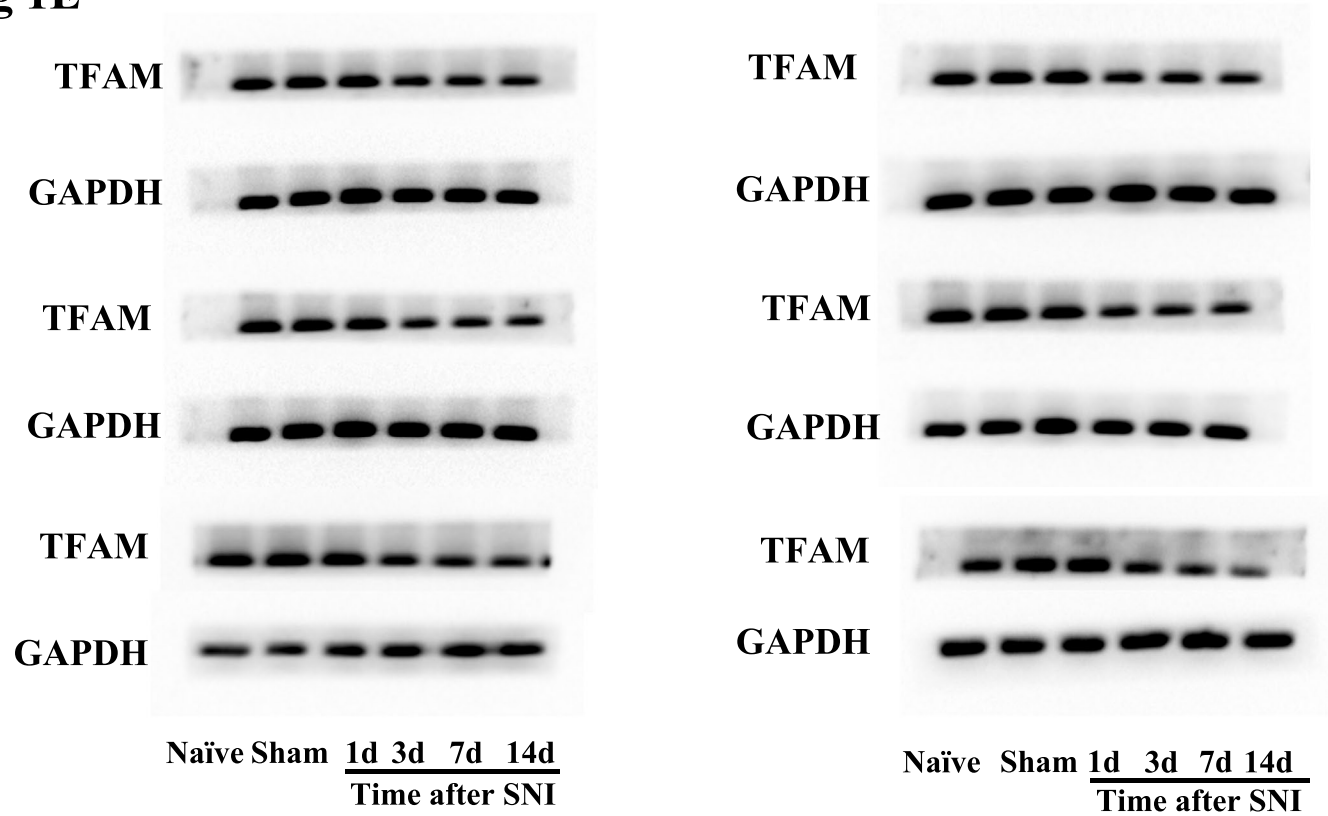

**Fig 3A**

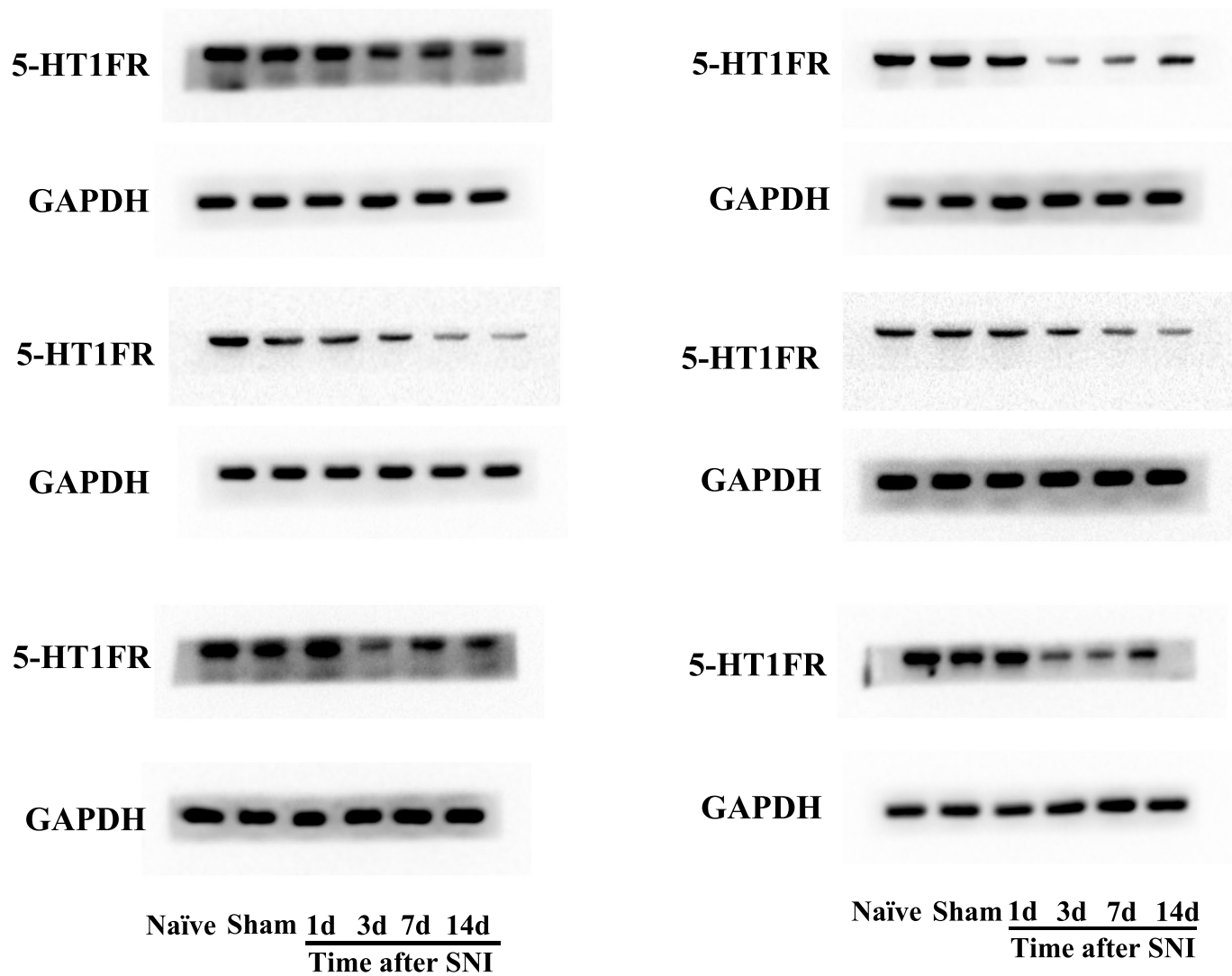

Supplement: Supplementary file 1 [file DataSheet1.ZIP › original blots/Fig. 1E, Fig. 3A.pdf]

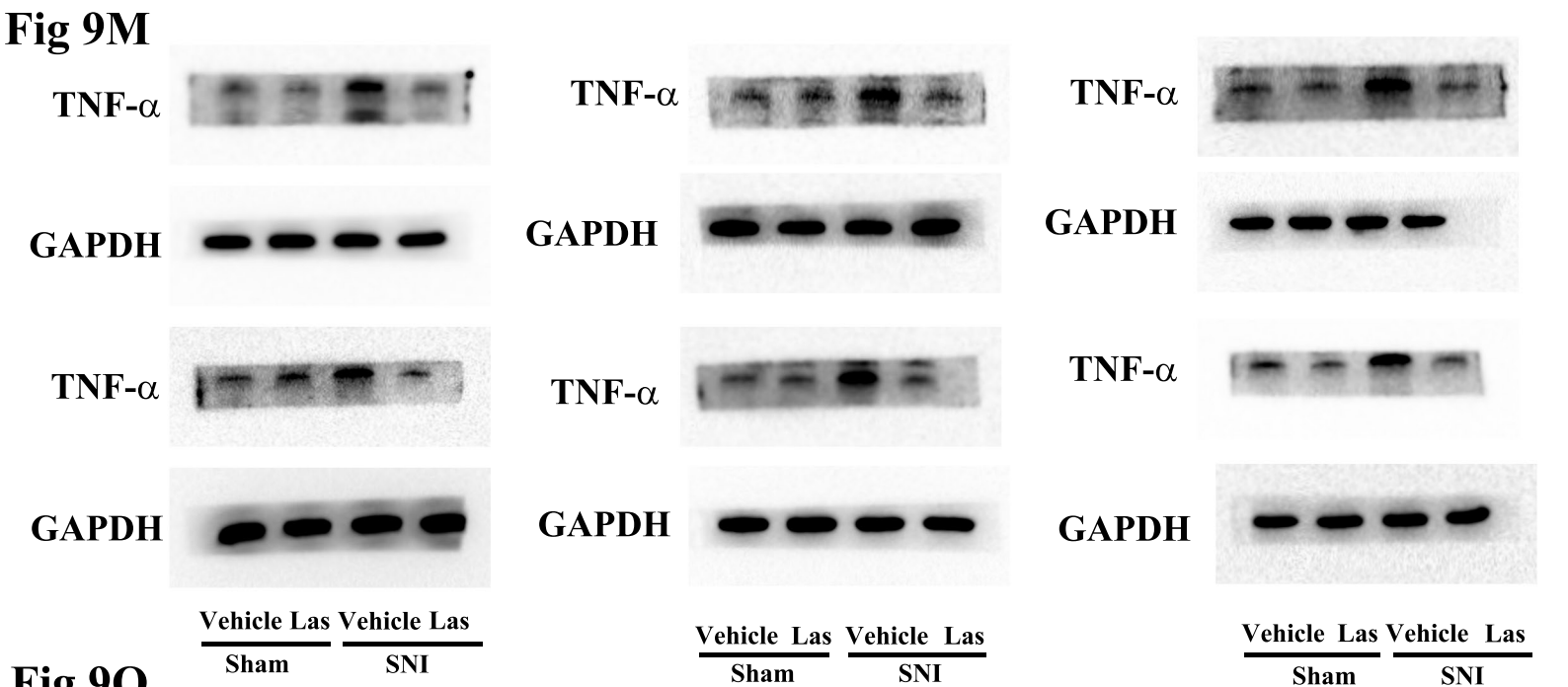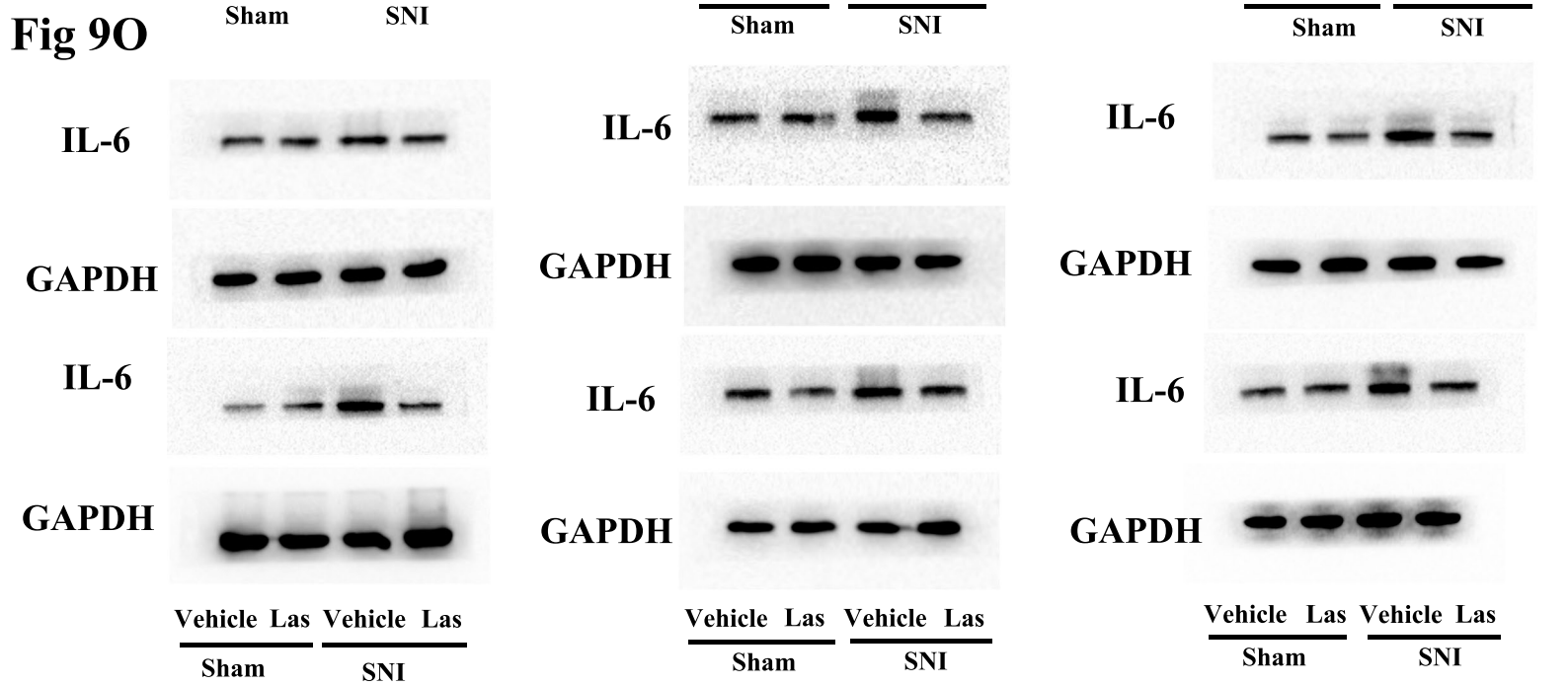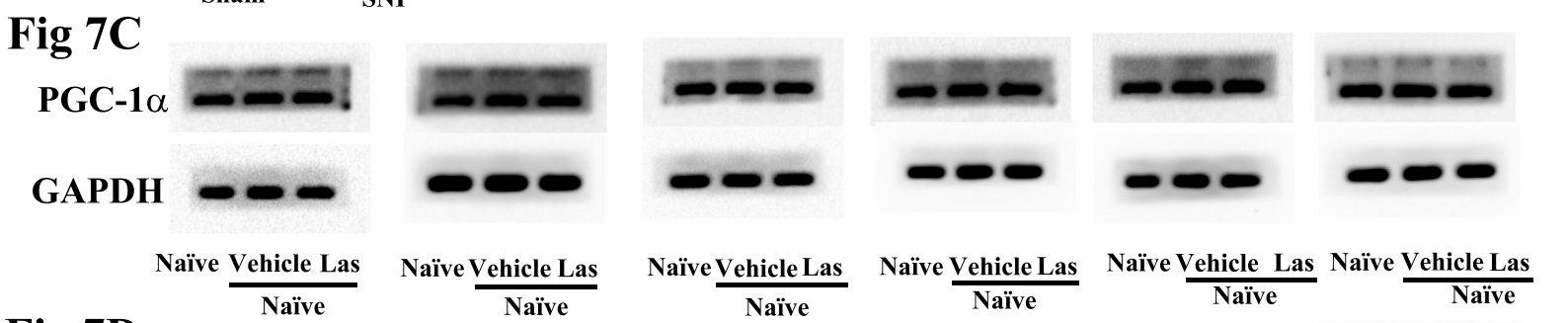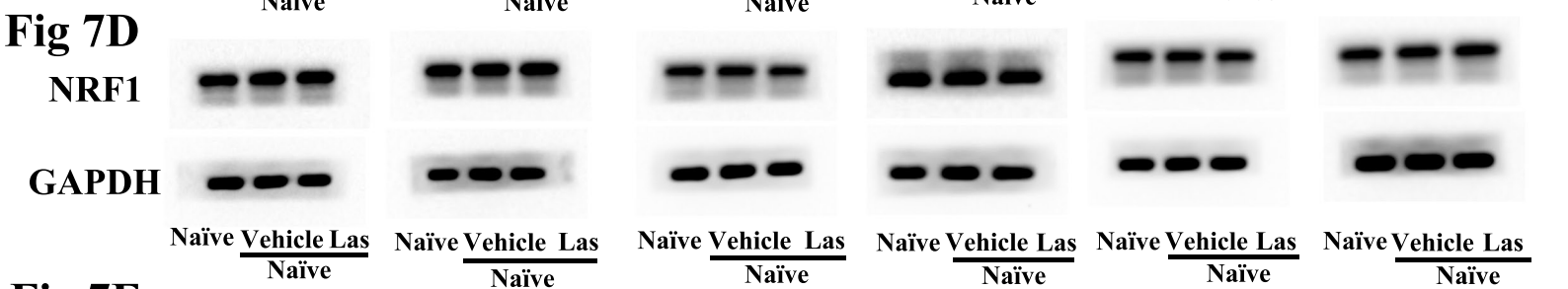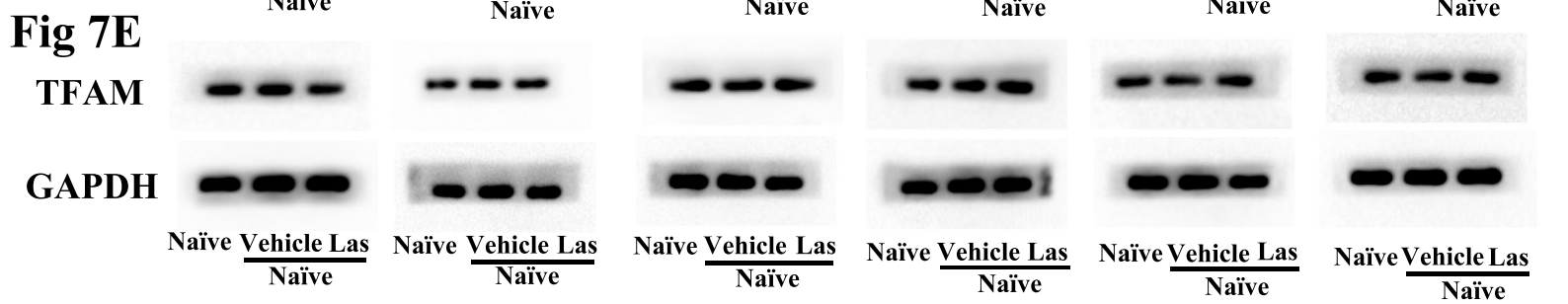

Supplement: Supplementary file 1 [file DataSheet1.ZIP › original blots/Fig. 7C-E and Fig. 9N-O.pdf]

**Fig 8B**

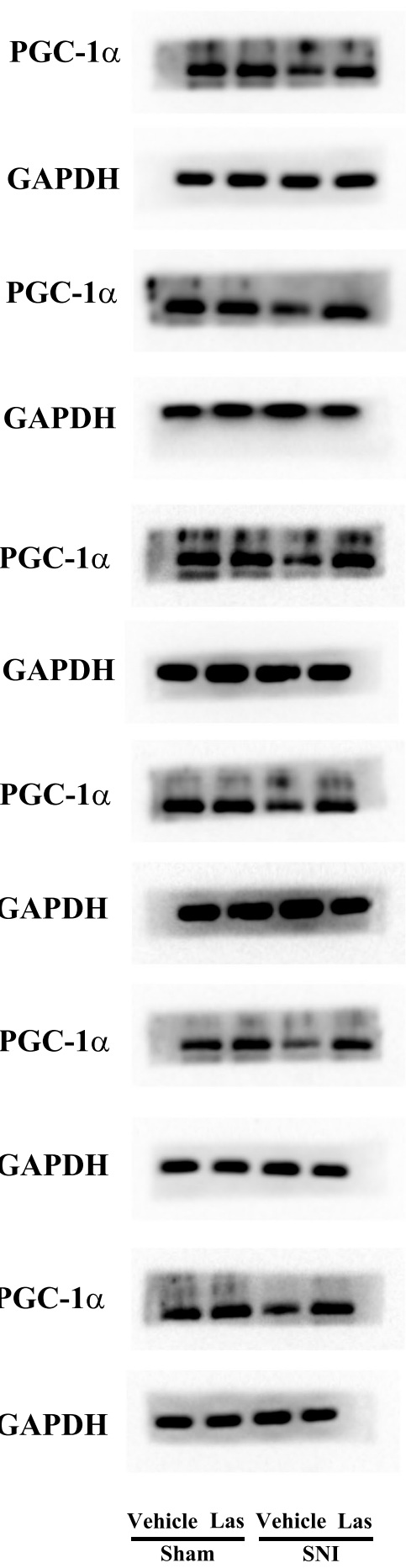

**Fig 8C**

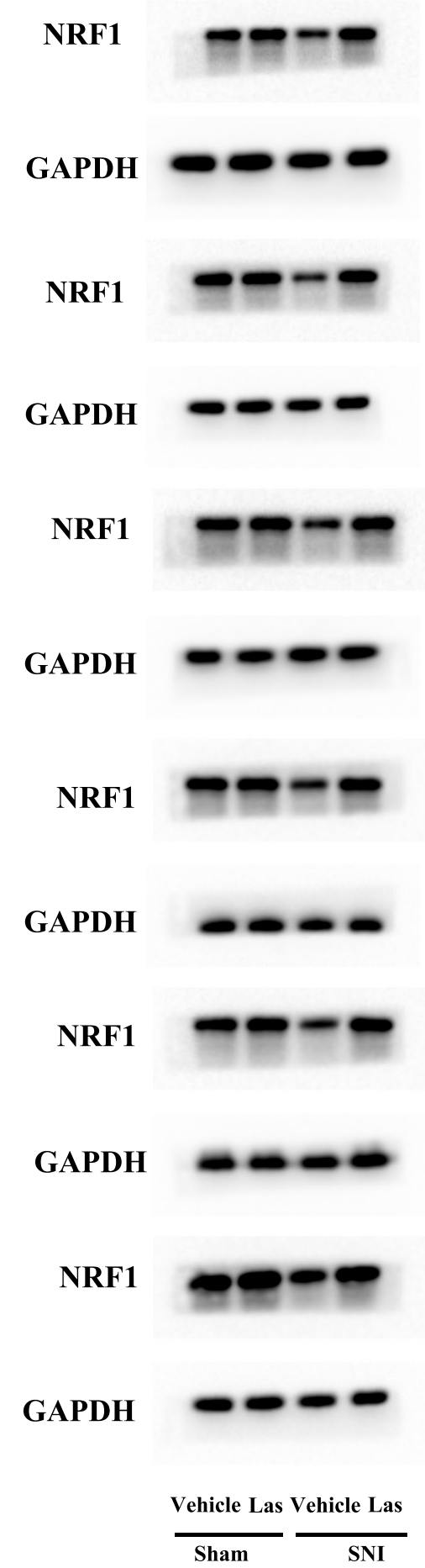

**Fig 8D**

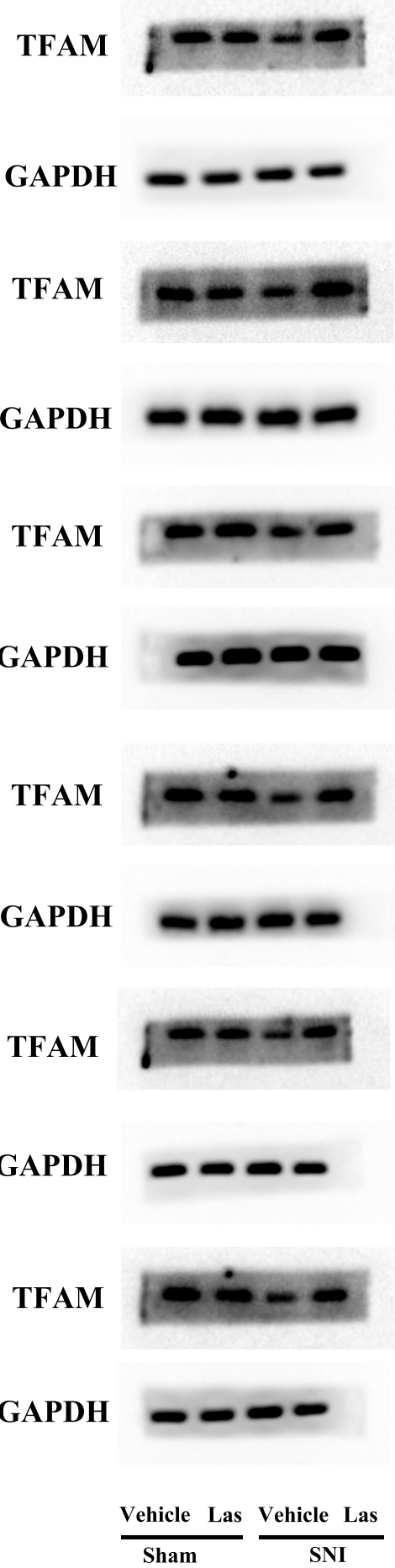

Supplement: Supplementary file 1 [file DataSheet1.ZIP › original blots/Fig. 8B-D.pdf]
